# Disrupting Cu trafficking as a potential therapy for cancer

**DOI:** 10.3389/fmolb.2022.1011294

**Published:** 2022-10-10

**Authors:** Zena Qasem, Matic Pavlin, Ida Ritacco, Matan Y. Avivi, Shelly Meron, Melanie Hirsch, Yulia Shenberger, Lada Gevorkyan-Airapetov, Alessandra Magistrato, Sharon Ruthstein

**Affiliations:** ^1^ Department of Chemistry and the Institute of Nanotechnology and Advanced Materials (BINA), Bar-Ilan University, Ramat-Gan, Israel; ^2^ National Research Council of Italy (CNR)—Institute of Material (IOM) C/o International School for Advanced Studies (SISSA), Trieste, Italy; ^3^ Department of Catalysis and Chemical Reaction Engineering, National Institute of Chemistry, Ljubljana, Slovenia; ^4^ Department of Chemistry, University of Salerno, Salerno, Italy; ^5^ The Mina and Everard Goodman Faculty of Life-Sciences, Bar-Ilan University, Ramat-Gan, Israel

**Keywords:** Atox1, ATP7B, cancer therapy, copper metabolism, peptide design

## Abstract

Copper ions play a crucial role in various cellular biological processes. However, these copper ions can also lead to toxicity when their concentration is not controlled by a sophisticated copper-trafficking system. Copper dys-homeostasis has been linked to a variety of diseases, including neurodegeneration and cancer. Therefore, manipulating Cu-trafficking to trigger selective cancer cell death may be a viable strategy with therapeutic benefit. By exploiting combined *in silico* and experimental strategies, we identified small peptides able to bind Atox1 and metal-binding domains 3-4 of ATP7B proteins. We found that these peptides reduced the proliferation of cancer cells owing to increased cellular copper ions concentration. These outcomes support the idea of harming copper trafficking as an opportunity for devising novel anti-cancer therapies.

## 1 Introduction

Copper is an essential trace element required for vital cell functions and the survival of all organisms, ranging from bacteria to humans (Burkhead et al., 2009; Gaggelli et al., 2009; Lutsenko, 2010). Owing to the ability of copper to cycle between two oxidation states [i.e., Cu(I) and Cu(II)], copper ions serve as important catalytic cofactors in protein-linked redox chemistry. Such proteins carry out fundamental biological functions and are required for proper cellular growth and development. Copper-dependent proteins are involved in a wide variety of biological processes. Therefore, copper deficiency in these enzymes and/or alterations in their functions often trigger disease states or physio-pathological conditions. Although critical for proper cell functioning, copper ions can also act as potent cytotoxins (Bertinato and L'Abbe, 2004; Manto, 2014; Harrison and Dameron, 1999). Owing to its special redox chemistry, copper readily participates in reactions that lead to the production of highly reactive oxygen species (ROS) (Culotta et al., 1999; Cerpa et al., 2005; Burkhead et al., 2009). Among these, hydroxyl radicals are thought to induce devastating cellular damage, including membrane lipid peroxidation, direct oxidation of proteins, and cleavage of DNA and RNA molecules. ROS generation is most likely the major contributing factor to the development of cancer and nervous system diseases and disorders, as well as aging. Hence, a sophisticated regulatory mechanism effectively limits the dynamic equilibrium underlying *in cell* copper concentrations (metal homeostasis) to very low levels and thus prevents copper-induced toxicity (Hunsaker and Franz, 2019; Spinello et al., 2021).

The main route for copper entry into mammalian cells is *via* the copper transporter Ctr1 (Eisses and Kaplan, 2002; De Feo et al., 2009; Walke et al., 2022), which is also responsible for shuttling the metal to constituents of different cellular pathways (Eisses and Kaplan, 2005; Maryon et al., 2013; Magistrato et al., 2019). Among these are the metallochaperone Atox1 and the ATP7A/B proteins in the Golgi apparatus, responsible for controlling Cu homeostasis. Mutations in ATP7A/B have been linked to copper deficiency (Menkes disease) and copper overload (Wilson disease) respectively. ATP7A and ATP7B are highly related in structure and function with approximately 60% amino acid identity. They comprise eight transmembrane segments, N-domain containing the ATP-binding site, P-domain containing the conserved aspartic acid residue, A-domain comprising the phosphate domain, and the N-terminal copper-binding domain (Forbes and Cox, 2000; de Bie et al., 2007; Banci et al., 2010a; Telianidis et al., 2013). The N-terminal domain is responsible for shuttling copper ions from Atox1 to the transmembrane domain of ATP7A/B (Walker et al., 2002). The N-terminal domain of ATP7A/B presents six metal-binding sub-domains (MBDs), each containing an MXCXXC motif known to coordinate Cu(I) *via* two cysteine residues (Badarau and Dennison, 2011; Shoshan et al., 2016). The MBDs of ATP7A/B share a high degree of structural similarity to the Atox1 monomer and are characterized by a ferredoxin-like fold with a compact βαββαβ structure (Banci et al., 2005; Banci et al., 2010b). Although the precise ATP7B MBD that interacts with Atox1 has been considered in several studies, which MBD is mainly responsible of Cu(I) trafficking remains controversial (Hussain et al., 2009; Yu et al., 2017; Magistrato et al., 2019; Qasem et al., 2019). For instance, other than MBD4, which has been suggested to be relevant for Cu(I) trafficking using spectroscopic experiments and molecular dynamics (MD) simulations, (Hussain et al., 2009; Qasem et al., 2019), other experimental efforts have suggested the involvement of MBD1-2 in the interaction with Atox1 (Badarau et al., 2010; LeShane et al., 2010; Yu et al., 2017; Zaccak et al., 2020).

Atox1 and MBDs of ATP7A/B are among the key regulators of copper trafficking and homeostasis maintenance. As such, they represent appealing targets for anti-cancer and neurological therapies. Recent knockdown studies of *Atox1* in cancer cells demonstrated that this copper chaperone is crucial for cancer cell proliferation and survival (Itoh et al., 2008; Beaino et al., 2014; Kim et al., 2019). Consistently, small molecules targeting Atox1 have been proved to effectively block Cu-trafficking and consequently reduce cell proliferation in lung, leukemia, breast, head, and neck cancer cell lines by elevating cellular ROS levels *via* Cu accumulation and reducing cellular NADPH and GSH levels. In contrast, healthy cells were minimally affected by these small molecules (Wang et al., 2015). Moreover, by inhibiting Cu(I) trafficking, it might also be possible to restore cancer cell sensitivity to Pt drugs, such as cisplatin, a widely employed anti-cancer drug used to treat selected solid tumors. Indeed, ATP7B plays an important role in resistance onset against Pt drugs, promoting their sequestration and excretion from cells, thus reducing their efficacy. It was also shown that by down-regulating levels of Atox1, which delivers Pt to ATP7B, Pt drug efflux was reduced, leading to increased Pt-induced cytotoxicity (Ishida et al., 2002; Boal and Rosenzweig, 2009; Larson et al., 2010; Theotoki et al., 2019).

Based on these findings, the use of small molecules to sabotage the ability of cells to maintain metal homeostasis may offer an opportunity to induce cancer cell death and/or re-sensitize resistant cells to metal drugs that can exploit copper trafficking routes (Weekley and He, 2017; Hunsaker and Franz, 2019). Building on atomic-level interactions between Atox1 and the MBD 3/4 units of ATP7B defined in earlier studies, we designed small peptides that can selectively interact with Atox1 and the MBDs of ATP7B (Qasem et al., 2019; Zaccak et al., 2020). After exhaustive *in silico* and experimental characterization of the binding modes of these peptides, we assessed their ability to induce selective breast and liver cancer cell death using cell viability and ^64^Cu(II) radio-labelling cell experiments. We showed that those peptides that interacted with both Atox1 and MBD 3/4 of ATP7B caused preferential toxicity in cancer cells. This may result in increased cellular copper concentrations, which becomes increasingly detrimental in cancer cells.

This study thus provides a proof-of-principle for the concept of disrupting Cu trafficking routes to the Golgi using peptides targeting distinct Cu chaperones as a viable and appealing strategy to preferentially trigger cancer cell death.

## 2 Results and discussions

### 2.1 Peptide design

With the aim of impairing Cu trafficking by blocking the Atox1/ATP7B trafficking route, we designed peptides that mimic the interactions of Atox1-MBD3/4 *in silico,* which were resolved in a previous study (Qasem et al., 2019). We hypothesized that although it is still controversial whether MBD3/MBD4 and, indeed, which MBDs preferentially interact with Atox1, a peptide targeting MDB3/MDB4 would nonetheless affect interactions of the different ATP7B domains and thus, the function of the ATP7B transporter. Initially, the seven residue-long stretch of MBD4 (SCVHSIE), and six residue-long portion of Atox1 (AEAVSR) that interact with the surface of the Atox1 and MBD4 protein structures, according to our previous MD studies (Qasem et al., 2019), were optimized by considering all possible mutations so as to increase binding affinity for the target proteins (Clark et al., 2019; Negron et al., 2019).

**FIGURE 1:**
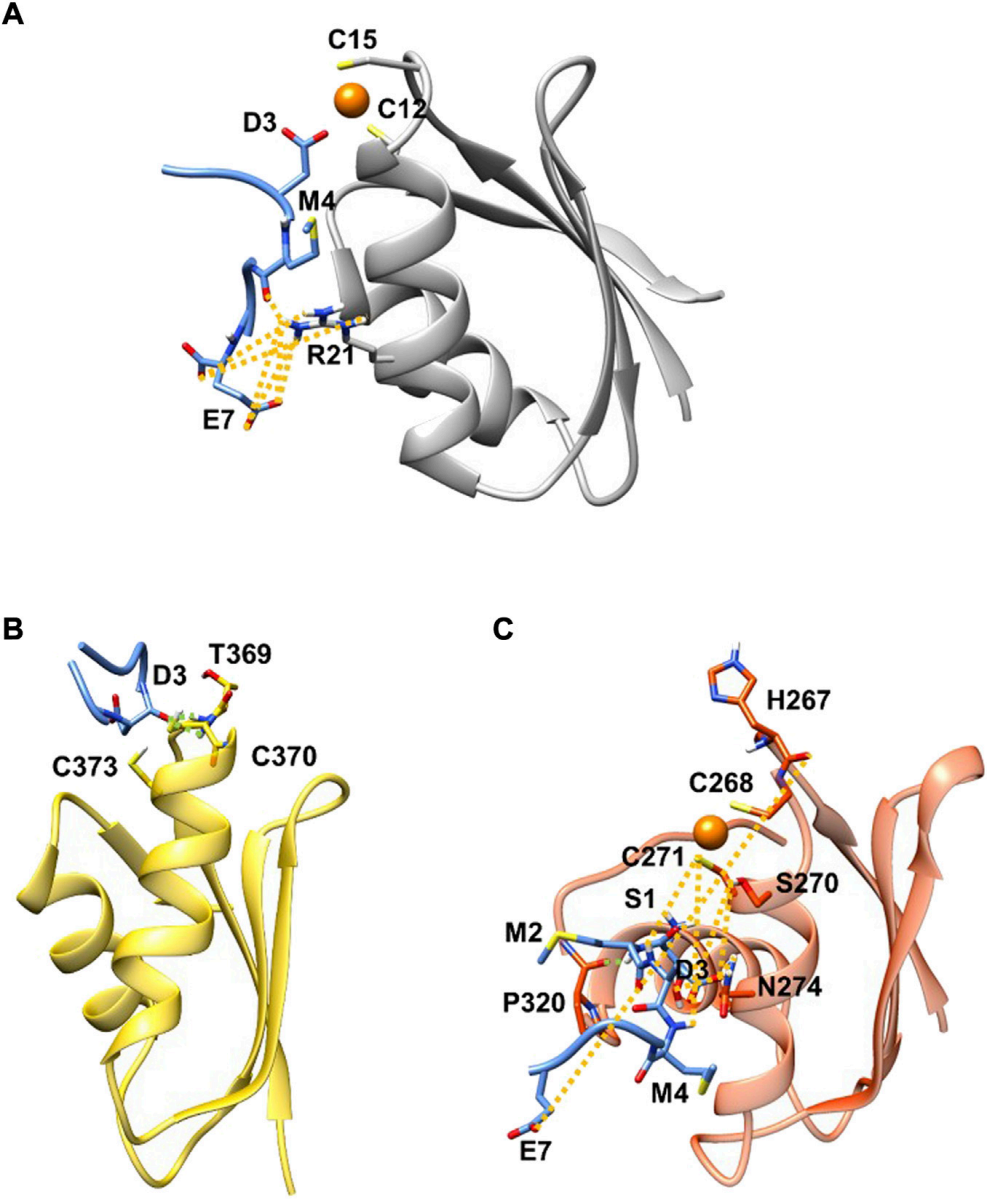
SMD (blue) in complex with Atox1 **(A)**(grey new cartoon), MBD4 **(B)** (yellow new cartoon), and MBD3 **(C)** (orange new cartoon). Hydrogen-bonds are denoted as dotted lines in the same colour scheme as in Supplementary Table S2. Oxygen atoms are shown in red, nitrogen in blue, sulphur in yellow, polar hydrogen atoms in white, copper in orange, and carbon atoms in the colour scheme of the protein. Non-polar hydrogen atoms are omitted for clarity.

**FIGURE 2:**
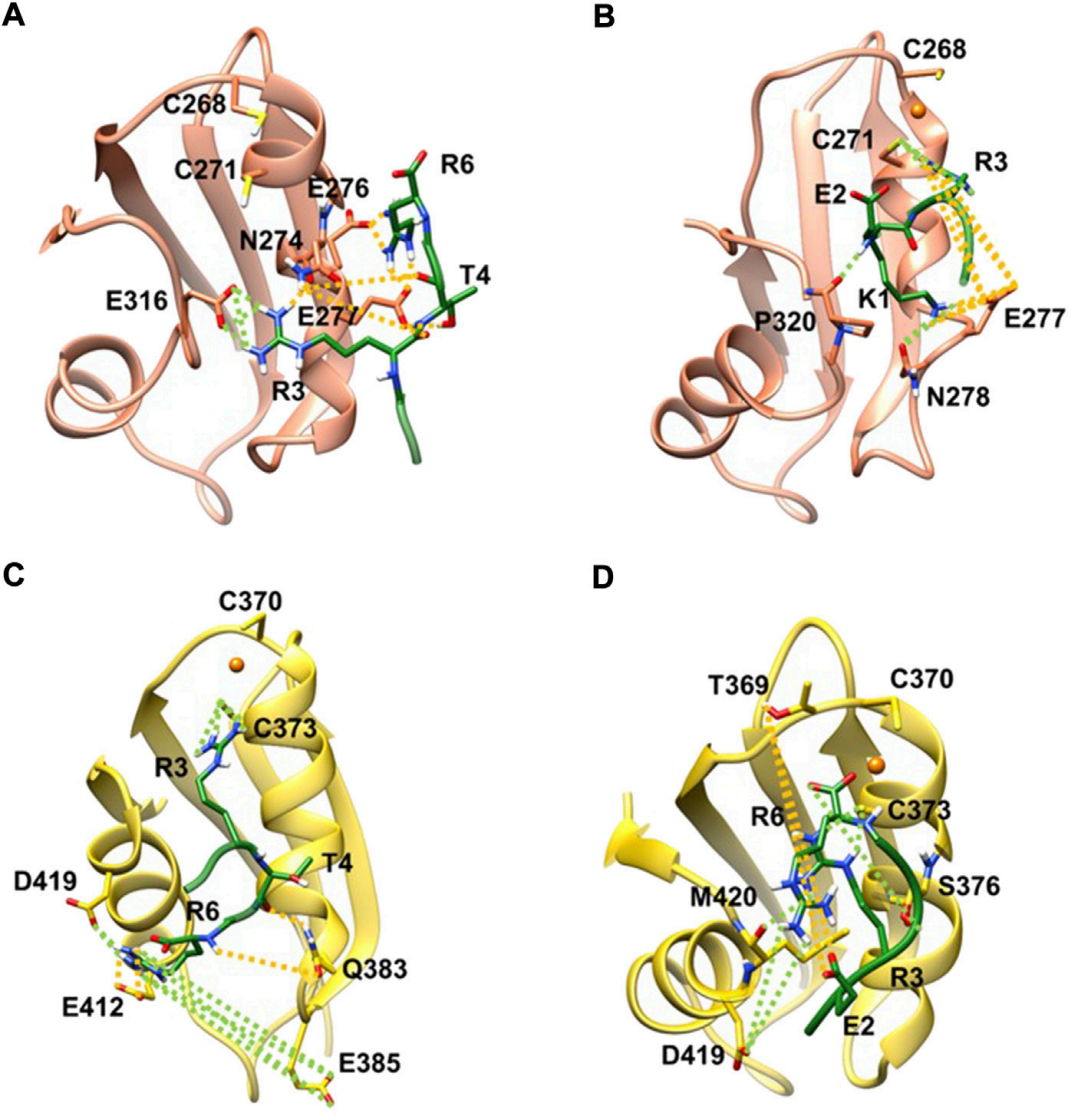
KER in complex with apo MBD3 **(A)**, (orange new cartoon), holo MBD3 **(B)** (yellow new cartoon), and holo MBD4 **(C, D)** (yellow new cartoon). Hydrogen-bonds are denoted as dotted lines in the same color scheme as in Supplementary Table S2. Oxygen atoms are denoted in red, nitrogen in blue, sulphur in yellow, polar hydrogen atoms in white, copper in orange, and carbon atoms in the color scheme of the protein. Nonpolar hydrogen atoms are omitted for clarity.

Among all possible variants, those peptides exhibiting the strongest calculated binding free energy (ΔGb) to Atox1 and MBD4 included a SMDMSIE sequence (hereafter referred to as SMD) and a KERTSR sequence (hereafter referred to as KER), respectively. After predicting their ability to penetrate cells in terms of cellular score and uptake efficiency (Manavalan et al., 2018), we performed classical MD simulations (200 ns-long) for the peptides in complex with the apo- and holo- (copper-bound) forms of the targeted proteins (Figures 1, 2, and Supplementary Table S1 in the supporting information (SI)) to verify their stability, to assess their binding mode, and to pinpoint key stabilizing interactions.

MD simulations suggest that SMD interacts with Atox1 through its Glu7 residue that forms a salt bridge interaction with Arg21 and Lys73@Atox1, whereas Asp3@SMD interacts with the metal (Figure 1A; Supplementary Table S2 in SI). Moreover, Met4@SMD establish hydrophobic interactions with Arg21@Atox1. This results in a ΔGb value of −22.7 ± 6.8 (Table 1) with Met4 and Ile6@SMD contributing the most to the binding free energy (Supplementary Table S3 in SI).

**TABLE 1:** Binding free energy (ΔGb, kcal/mol) of the SMD and KER peptides to Atox1 and MBD 3/4 of ATP7B, respectively, obtained with the Molecular Mechanics/Generalized Born Surface Area (MM-GBSA) method. The electrostatic and Van der Waals components of the binding free energy are reported. Cu(I) indicates whether the protein is simulated in the presence (x) or absence (-) of a bound Cu(I) ion. Standard deviation is reported. In this table, only the ΔGb of the peptide/target models that were stable during molecular dynamics simulations are reported. A complete list of all models built and simulated is reported in Supplementary Table S1.

Adduct	Cu(I)	Electrostatic	vdW	ΔGb
Atox1-SMD	x	−201.6 ± 67.9	−23.3 ± 7.1	−22.7 ± 6.8
MBD3-SMD	x	−29.6 ± 55.3	−27.1 ± 6.1	−10.3 ± 9.2
MBD4-SMD	-	174.0 ± 28.9	−22.3 ± 8.1	−17.1 ± 7.4
MBD3-KER	-	−248.3 ± 84.6	−21.9 ± 6.6	−22.2 ± 7.8
MBD3-KER	x	−303.8 ± 43.5	−19.8 ± 5.9	−18.5 ± 6.4
MBD4-KER (pose1)	x	−467.8 ± 37.2	−26.3 ± 4.8	−30.1 ± 5.0
MBD4-KER (pose2)	x	−443.6 ± 65.7	−18.5 ± 5.3	−21.8 ± 5.9

Next, we investigated the ability of SMD to bind the two ATP7B MBDs. We found that, SMD formed stable interactions with apo MBD4 (ΔGb of −17.1 ± 7.4 kcal/mol), forming persistent H-bonds between Asp3@SMD to Thr369 and Cys370@MBD4 (Figure 1B; Supplementary Table S3 in SI). In addition, Met2, Met4, and Ile6@SMD established hydrophobic interactions with the target proteins, which contributed to stabilizing the binding of the peptide. SMD also interacted with holo-MBD3 (ΔGb of −10.3 ± 9.2 kcal/mol), with the interaction being stabilized by multiple contacts that primarily involve the SMD Glu7, Asp3, and Ser1 and Met2 residues (Figure 1C and Supplementary Tables S2, S3 in SI).

We then explored the binding of KER peptide originally designed to interact with ATP7B MBD4 and found to indeed bind the holo-form of MBD4. This was observed in two independent trajectories provided by two different docking poses of the peptide. In both forms, the strongest interactions were provided by H-bond pairs Cy373@MBD4-Arg3@KER and Asp419@MBD4-Arg6@KER In addition, in one pose, H-bonds are also formed between Glu385 and Glu412@MBD4 with Arg6@KER, whereas in the second structure Ser376@MBD4 H-bonds with Arg6@KER and Met420@MBD4 H-bonds with Arg3@KER. The highest ΔGb values of −30.1 ± 5.0 kcal/mol corresponded to the first docking pose. KER also strongly bound to MBD3, although it bound stronger to the apo-than holo-form of the protein (ΔGb values of −22.2 ± 7.8 and −18.5 ± 6.4 kcal/mol for the apo- and holo-forms, respectively). The strongest interactions were provided by H-bonds formed between Asn274, Glu276, Glu277, and Glu 316@MBD3 with Arg3, Thr4, and Arg6@KER in the apo-form, while in the holo-form, the strongest interactions were due to H-bonds between Cys271, Glu277, Asn278, and Pro320@MBD3 and Lys1, Glu2, and Arg3@KER (Figures 2A,B and Supplementary Table S3 in SI).

In summary, SMD appear to be multi-targeting compound that can interact with both Atox1 and MDB3/4, while KER is predicted to target only MBD3/4.

### 2.2 *In vitro* characterization of Atox1 and MBD3/4 interactions with the SMD and KER peptides

Based on these findings, we proceeded to synthesize and characterize the two peptides. The KER and SMD peptides were synthesized at a 99.4% and 95.1% purity, respectively (Supplementary Figures S1, S2 and Supplementary Table S4 in the SI). To confirm and characterize the interactions between the targeted proteins and the designed peptides predicted by all-atom simulations, we performed electron paramagnetic resonance (EPR) spectroscopy, circular dichroism (CD), and microscale thermophoresis (MST) experiments.

Continuous wave (CW) EPR experiments can provide insight into protein dynamics at room temperature in solution. The combination of CW-EPR with site-directed spin labeling, (Columbus and Hubbell, 2002; Hubbell et al., 2013; Jeschke, 2013), wherein an electron spin is introduced into diamagnetic proteins, can provide information on the local environment of such proteins and the mobility of protein domains (Columbus and Hubbell, 2002; Levy et al., 2014a; Shenberger et al., 2015). CW-EPR can thus be used to investigate the binding interactions of proteins with small-molecules/protein partners. To explore the binding interactions of MBD3-4 and Atox1 with the two peptides (KER and SMD), MBD3-4 was spin-labeled with the methanesulfonothioate (MTSSL) nitroxide radical at the native cysteine residues C305 (located in the loop between β3 and α2 of MBD3), C358, and C431 (located in the loop between β1 and β4 of MBD4), whereas Atox1 was spin-labeled at C41. These spin labelling positions were already extensively employed in previous studies (Qasem et al., 2019). The CW-EPR spectra of labelled MBD3/4 or Atox1 were not affected by the presence of the KER and SMD peptides (Supplementary Figure S3 in SI). Only minor changes occurred to MBD3-4 in the presence of Cu(I) and the peptides. As such, the CW-EPR spectra suggested that both peptides weakly interacted with MBD3-4 and/or Atox1 or that they interacted with the proteins at sites distant from the spin labels, thus rendering the CW-EPR signal insensitive to their presence.

We then performed double electron electron resonance (DEER) experiments (Supplementary Figure S3 in SI) on spin-labeled MBD3-4 in the presence of non spin-labeled Atox1 and the KER and SMD peptides. DEER experiments can estimate the distance distributions between spin-labels (Schiemann et al., 2021). In the absence of the peptides, a single distribution of 1.9 ± 0.2 nm was obtained. After addition of SMD peptide to the solution, this distribution shifted to 2.0 ± 0.2 nm, while in the presence of KER peptide, the distribution was at 1.8 ± 0.2 nm. The DEER experiments suggested that minor conformational changes occurred in MBD3-4 in the presence of Atox1 and the KER and SMD peptides. Moreover, they revealed that these changes differed in the presence of KER or SMD peptide.

Complementarily CD measurements revealed that the peptides only slightly affected the secondary structure of the proteins (Supplementary Figure S4 in SI). The largest changes were observed for MBD3/4 in the presence of SMD peptide, whereas they were more modest in the presence of KER peptide. Atox1 secondary structure was only slightly affected by the SMD peptide and was unaffected by the KER peptide.

**FIGURE 3:**
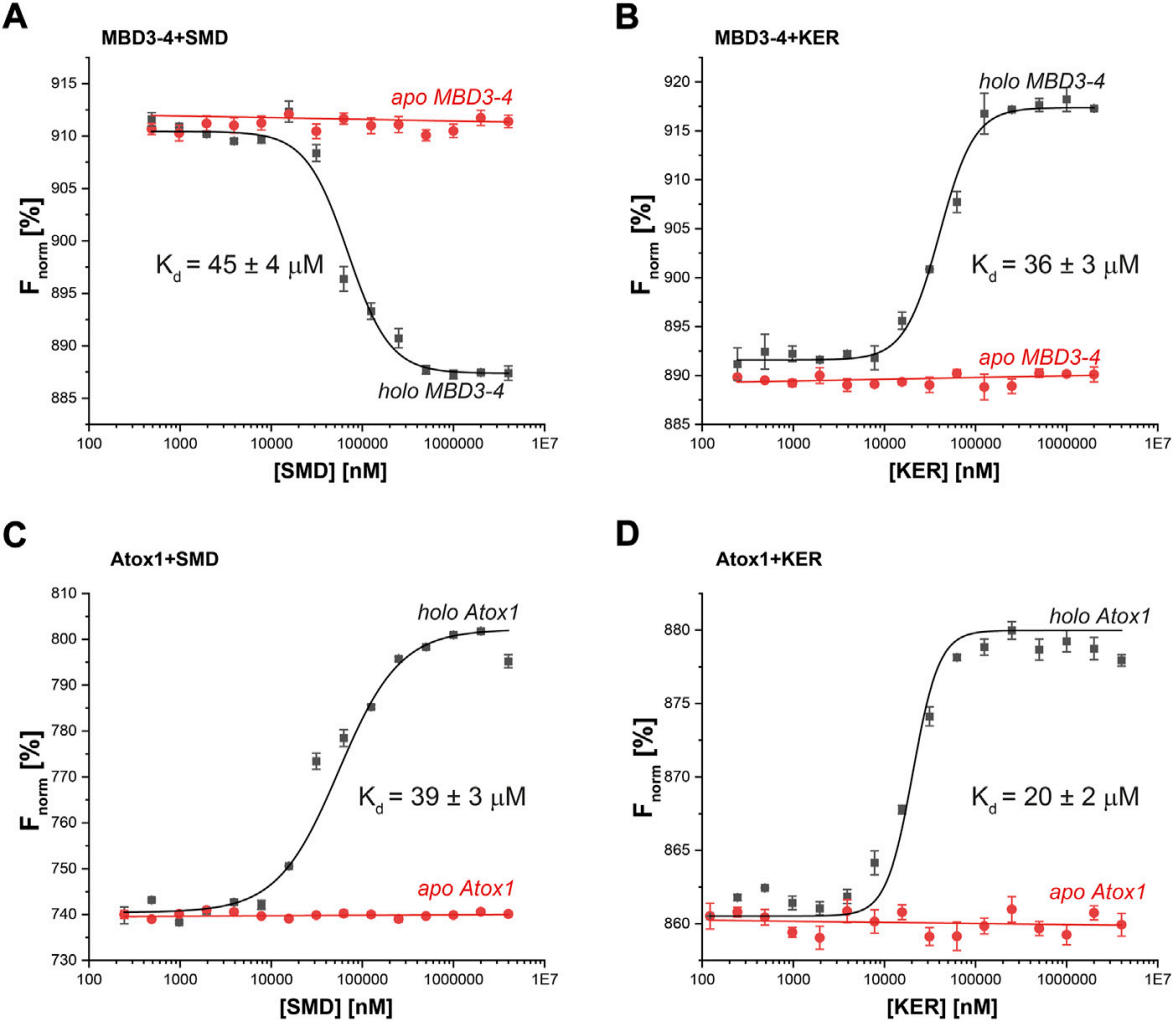
Thermophoresis analysis of protein-peptide interactions in the presence (black curves) or absence (red curves) of Cu(I) (40 µM) for **(A)** MBD3-4 protein and SMD peptide; **(B)** MBD3-4 protein and KER peptide; **(C)** Atox1 protein and SMD peptide; **(D)** Atox1 protein and KER peptide. The binding isotherms were generated by plotting normalized fluorescence against the concentration of a non-fluorescent binding partner (peptide). The protein concentration was 50 nM. Error bars were calculated based on three independent measurements.

To better understand the EPR and CD experiments, MST binding experiments were performed. MST is a physical phenomenon whereby biomolecules migrate along a temperature gradient according to their size, hydrodynamic radius, charge, and hydration shell. The target biomolecule is fluorescently labeled and monitored over the course of the experiment. An infra-red laser is used to create a temperature gradient for a given time. Since binding interactions can alter the properties listed above, a binding curve can be created with a ligand titration series. Fixed amounts of labelled MBD3-4 or Atox1 were incubated with increasing concentrations of SMD or KER peptides, in the presence or absence of Cu(I) (Figure 3). Strikingly, the results indicated that the interaction between MBD3-4, Atox1, and the two peptides occurred exclusively in the presence of Cu(I). The measured K_D_ values of the SMD peptide were in the tens of µM range for both Atox1 (39 ± 3 μM) and MBD3-4 (45 ± 4 μM), whereas KER peptide preferentially interacted with Atox1 (20 ± 2 μM). The K_D_ for the interaction with MBD3-4 was slightly higher (36 ± 3 μM). Interestingly, an opposite thermophoretic behavior was observed for MBD3-4 in the presence of SMD and KER peptides, but not of Atox1. As such, while MD simulations predicted the relative binding affinity to the target for the SMD peptide, they are not able to find a stable binding pose for KER peptide binding to Atox1. Considering the close binding energy for the two interactions, these data suggest that SMD and KER induced different conformations of MBD3-4 with similar affinity (Moon et al., 2018). This observation supports the CD data indicating that SMD induced secondary structural changes to MBD3-4.

Finally, we also performed MST experiments in the presence of a control peptide (GAGAAA, termed Ala) to further assess the reliability of our results. No binding interaction was observed with MBD3-4 and Atox1, neither in the presence nor in the absence of Cu(I) (Supplementary Figure S5 in SI). We also checked the interaction between the KER and SMD peptides in the presence of another protein, CueR, an *Escherichia coli* copper receptor that also coordinates a Cu(I) ion through a thiolate bridge (Changela et al., 2003; Sameach et al., 2017). No interaction between the peptides and the CueR protein was detected (Supplementary Figure S6 in SI), confirming the specificity of the binding of the KER and SMD peptides to Atox1 and the MBDs of ATP7B.

Taken together, these *in vitro* experiments suggest that both SMD and KER can work as dual-targeting peptides through the Atox1/ATP7B Cu-trafficking route. Owing to the similarity of ATP7B and ATP7A, these peptides can, in principle, also interact with MBDs of ATP7A.

### 2.3 SMD and KER peptides reduce cell viability in a copper-dependent manner

**FIGURE 4:**
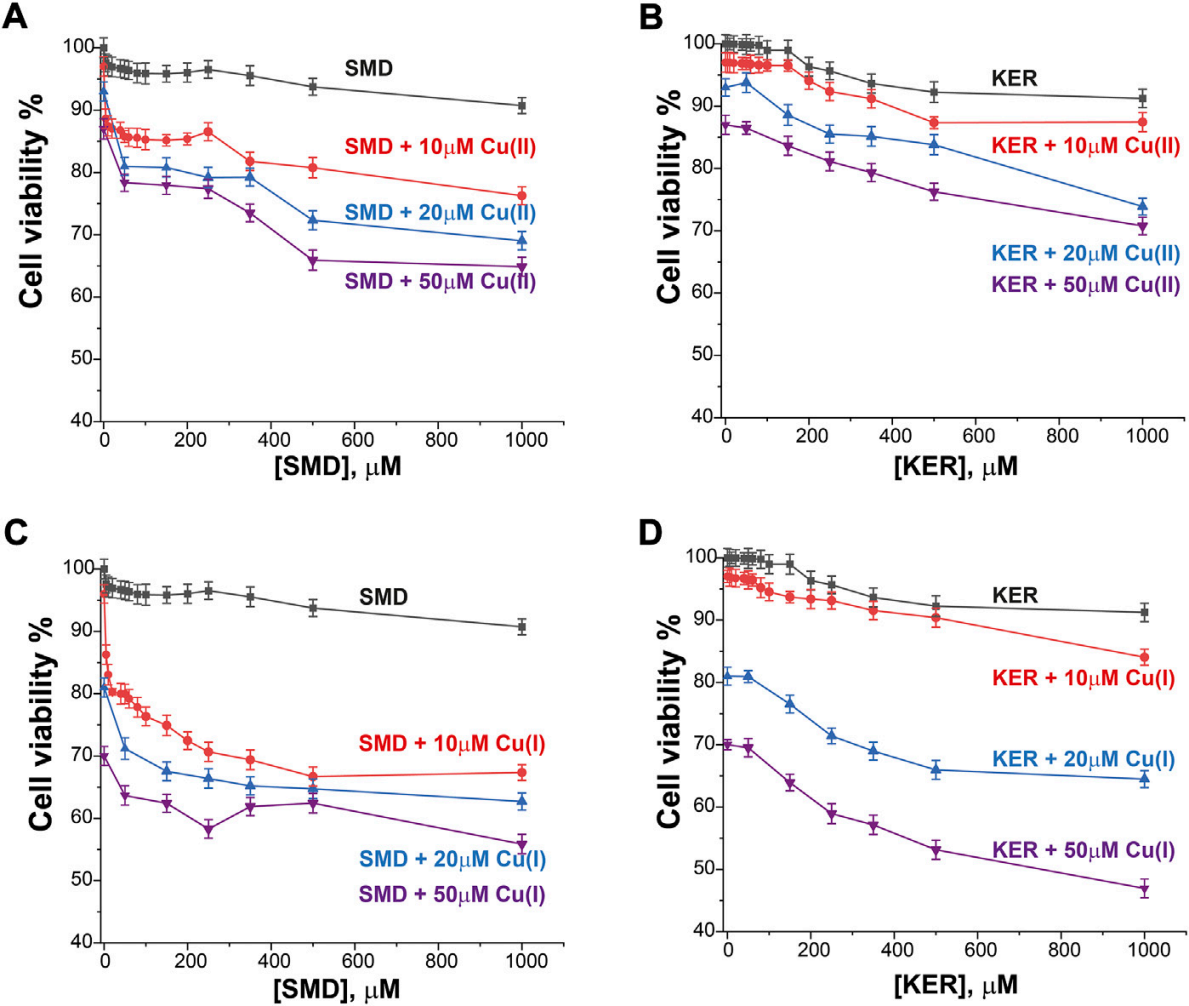
HEK293 cell viability in the presence of SMD peptide at varying Cu(II) and Cu(I) concentrations in **(A)** and **(C)**, respectively, and in the presence of KER peptide at varying Cu(II) and Cu(I) concentrations **(B)** and **(D)**, respectively. At [peptide] = 0, cell viability in the presence of [Cu(II)/Cu(I)] is given. Error bars were calculated based on three independent measurements.

**FIGURE 5:**
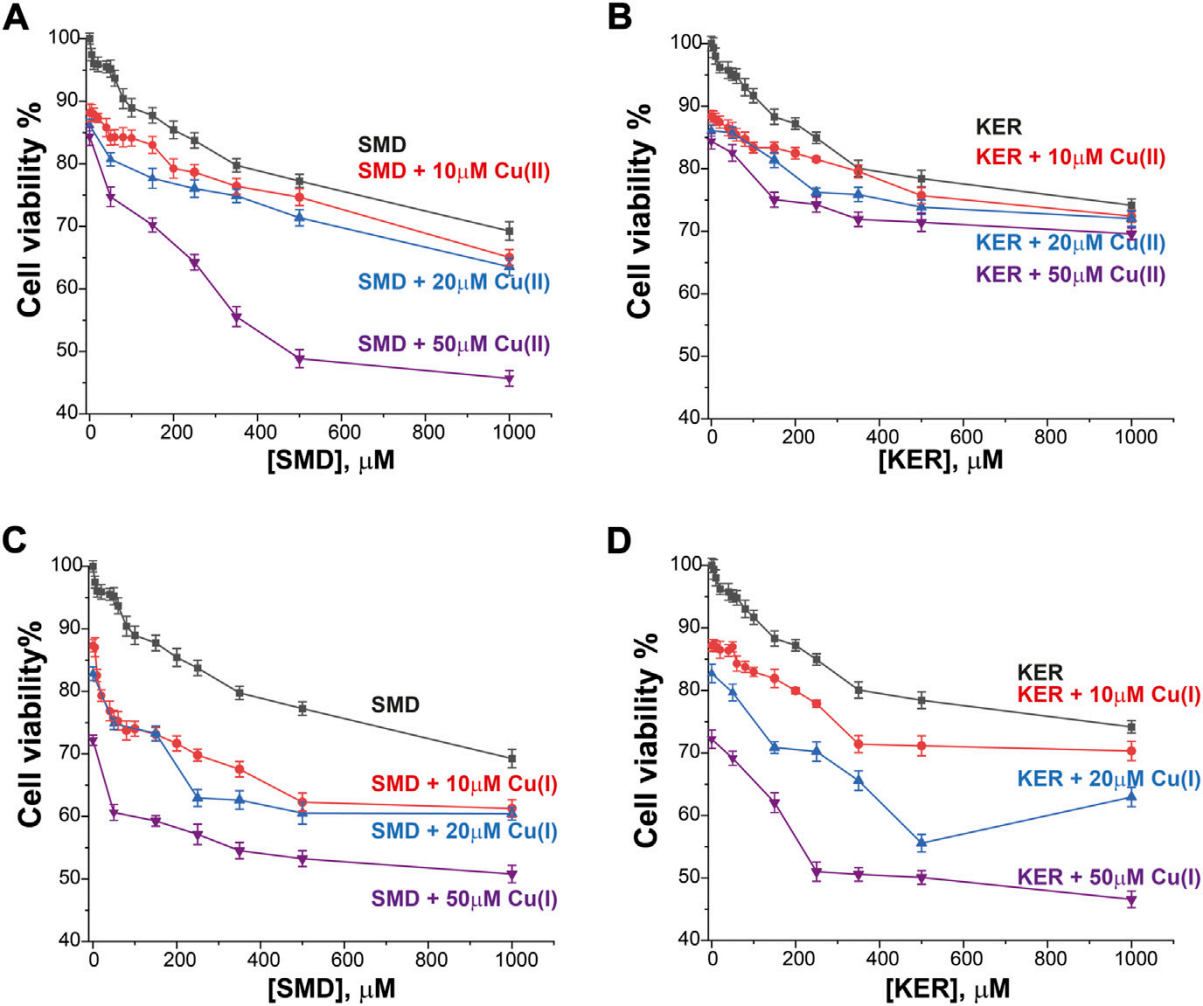
MCF7 breast cancer cell viability in the presence of SMD peptide at varying Cu(II) and Cu(I) concentrations in **(A)** and **(C)**, respectively, and in the presence of KER peptide at varying Cu(II) and Cu(I) concentrations in **(B)** and **(D)**, respectively. At [peptide] = 0, cell viability in the presence of [Cu(II)/Cu(I)] is given. Error bars were calculated based on three independent measurements.

**FIGURE 6:**
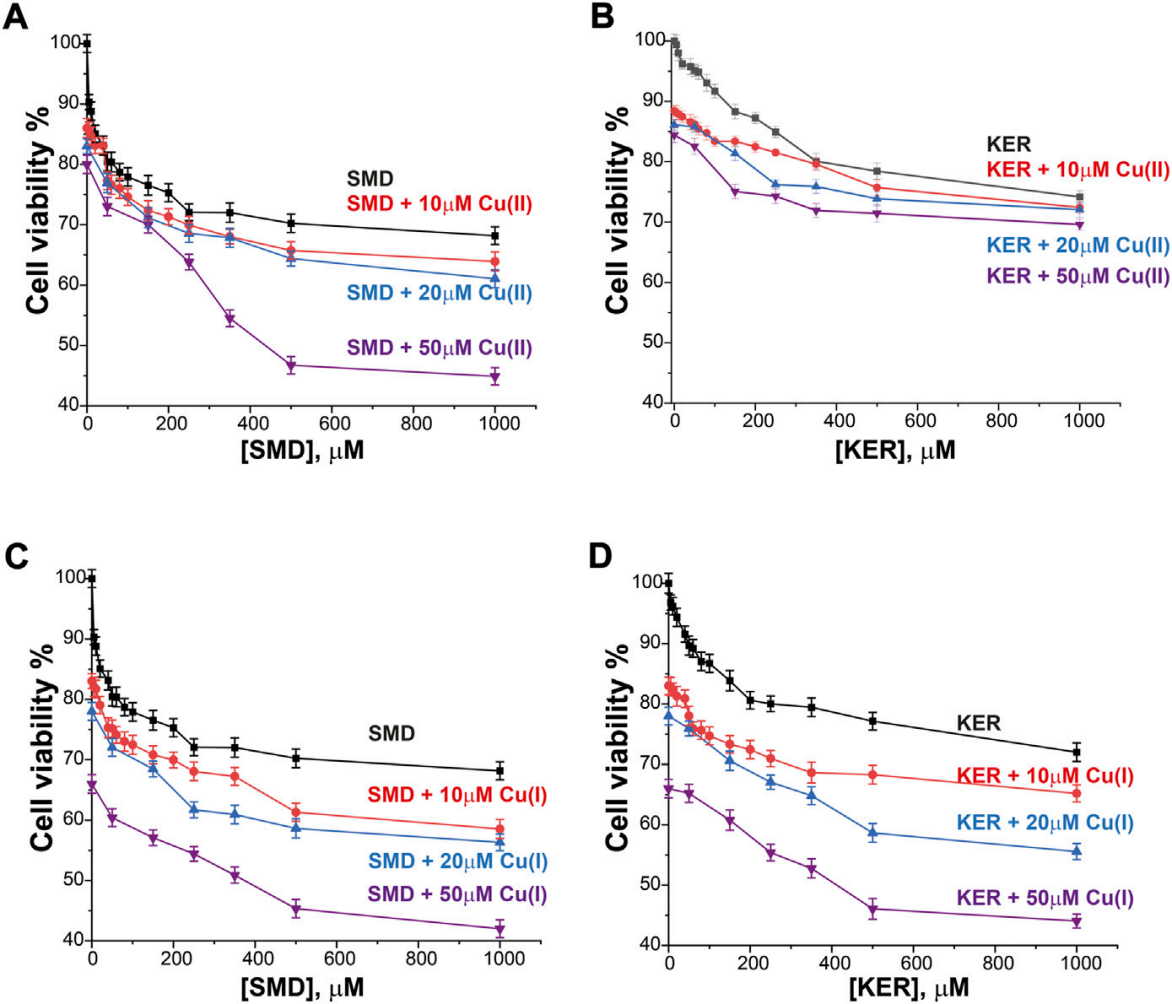
HepG2 liver cancer cell viability in the presence of SMD peptide at varying Cu(II) and Cu(I) concentrations in **(A)** and **(C)**, respectively, and in the presence of KER peptide and varying Cu(II) and Cu(I) concentrations in **(B)** and **(D)**, respectively. At [peptide] = 0, cell viability value in the presence of [Cu(II)/Cu(I)] is given. Error bars were calculated based on three independent measurements.

To assess the potential of these peptides in cancer therapy, we investigated their cytotoxic effects on three cell lines, HEK293 human kidney cells (Figure 4), MCF7 breast cancer cells (Figure 5), and HepG2 liver cancer cells (Figure 6) as proxies of healthy and cancer cells. Initially, cell viability experiments were conducted in the presence of Cu(II) and Cu(I) ions (in the absence of peptides). We observed that the cells (Supplementary Figure S7, SI) remained viable until high copper concentrations were reached, irrespective of the redox state (in HEK293 cells, the LC_50_ for Cu(II) was 196 μM and for Cu(I) it was 150 μM; in MCF7 cells, the LC_50_ for Cu(II) was 180 μM and for Cu(I) it was 165 μM; in HepG2 cells, the LC_50_ for Cu(II) was 205 μM and for Cu(I) it was 170 μM). We then assessed peptide cytotoxicity in the absence of copper and at different copper concentrations (i.e., [Cu(II)] and [Cu(I)] = 10, 20, and 50 μM). In the absence of copper, the viability of the HEK293 cells was essentially unaffected by the peptides, whereas in the MCF7 cancer cells, the peptides decreased cell viability by 10–20%, at a peptide concentration of 200 µM. In HepG2 cancer cells, the peptides decreased cell viability by 20–25%, at a peptide concentration of 200 µM. Conversely, in the presence of copper (even at a [Cu(I/II)] of 10 μM), cytotoxicity of the peptides increased, supporting our hypothesis that mechanism of action of the peptides involves an alteration of copper homeostasis. The SMD peptide was slightly more toxic than the KER peptide. In the presence of 50 μM SMD and 10 μM Cu(I), there was a 25% reduction in cell viability of MCF7 and HepG2 cells and a 20% reduction in HEK293 cell viability. In the presence of 50 μM of KER and 10 μM Cu(I), there was a 15% reduction in MCF7 and HepG2 cell viability and a 3% reduction in HEK293 cell viability. These effects suggest the preferential lethality of the peptides for cancer cells. A higher concentration of peptides still exhibited preferential cytotoxicity for MCF7 and HepG2 cells, as compared with HEK293 cells. Nevertheless, only an extremely high concentration of the SMD peptide (500 μM at a [Cu(II)] of 50 µM) triggered a 50% reduction in the viability of MCF7 cells, a 55% reduction in HepG2 cell viability, and a 40% drop in HEK293 cell survival. Under the same conditions, the KER peptide reduced the viability of MCF7, HepG2, and HEK293 cells by 30%, 55%, and 25%, respectively.

### 2.4 SMD and KER peptides enhance Cu uptake by cancer cells

**FIGURE 7:**
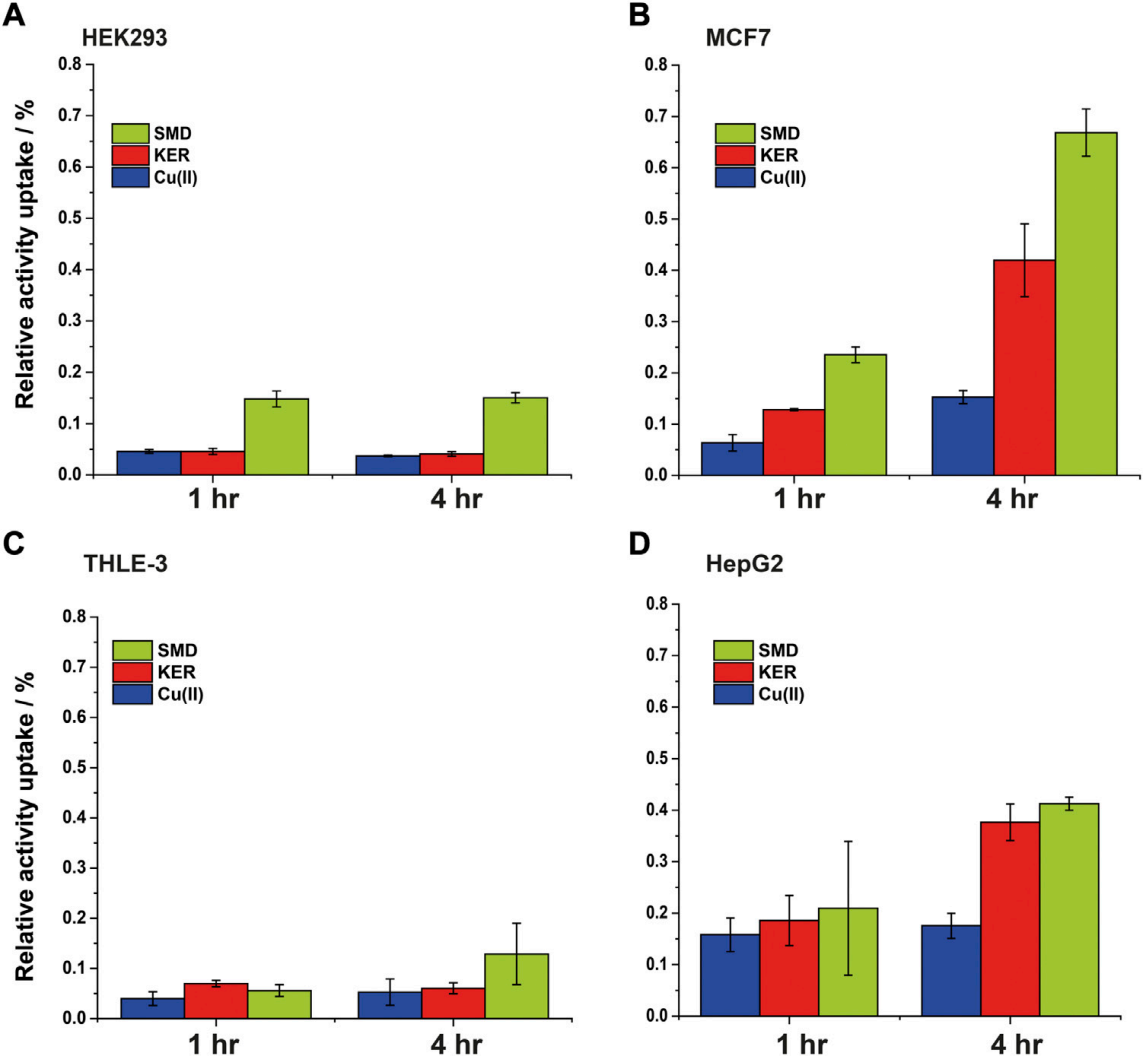
Intracellular ^64^Cu content in **(A)** HEK293 cells **(B)** MCF7 cancer cells, **(C)** THLE-3 liver cells, and **(D)** HepG2 liver cancer cells. Error bars were calculated based on three independent measurements.

Radiolabeled ^64^Cu(II) uptake experiments were performed to investigate the effect of the SMD and KER peptides on copper metabolism in cells. The size of the radio-isotope signal was plotted as the relative activity uptake (%) of radioactive material in cell cultures over time (Figure 7). In the absence of peptides, ^64^Cu uptake by MCF7 and HepG2 cells was slightly higher than by HEK293 cells. This is similar to what was reported in our previous study showing that the copper uptake was higher in cancer cells, as compared to healthy human cells (Walke et al., 2021). To confirm this result, we also employed THLE-3 liver epithelial cells as an example of human healthy cells. These showed similar uptake as did HEK293 cells. In the presence of the KER and SMD peptides, radiolabeled ^64^Cu uptake by MCF7 and HepG2 cancer cells was enhanced, relative to healthy HEK293 and THLE-3 cells. This further suggests that the peptides altered Cu trafficking. The most prominent effect was detected for SMD peptide in MCF7 cells, where a five-fold rise in intracellular copper concentration was induced 4 h after ^64^Cu(II) injection, as compared to the three-fold increase induced by the KER peptide. This is consistent with the observation that the SMD peptide was slightly more toxic to the cells than was the KER peptide. The disruption of copper homeostasis by the peptides increased copper accumulation in the cell, which led to toxicity. In order to understand whether the different copper uptake ratio in cancer cells is related to the Ctr1 expression level, we performed western blot for the various cell lines (Supplementary Figure S8, SI). The experiments suggest that in cancer cells the expression level of Ctr1 is a bit lower or in the same level of normal cells, but it is not upregulated. Therefore, the higher copper content in cancer maybe be either related to downregulation of ATP7A/B or upregulation of intracellular copper proteins. Recent study (Blockhuys et al., 2017) explored the gene expression levels of all proteins that are involved in copper metabolism in 18 different cancer types and compared it to normal cells. They found out that in breast cancer cells, Atox1 and ATP7B are upregulated, while Ctr1 is a bit downregulated. However, in liver cancer cells, only Atox1 is upregulated. Therefore, the higher accumulation of copper in cancer cells is probably owing to higher expression level of Atox1.

## 3 Conclusion

Copper metabolism is central to cancer, as overwhelmingly proven by several studies (Ishida et al., 2002; Itoh et al., 2008; Blockhuys et al., 2020). Cancer cells are known to accumulate copper ions more than healthy cells. Indeed, elevated copper levels have been reported in the case of different solid tumor tissues, suggesting that copper dys-homeostasis plays a critical role in cancer progression and migration (Ge et al., 2022). As such, targeting Cu-trafficking could represent a novel strategy for combatting cancer.

Among the players acting in the Cu-trafficking system, Atox1 appears to play a central node. This protein affects distinct Cu delivery routes implicated in cancer cell migration (Blockhuys and Wittung-Stafshede, 2017; Blockhuys et al., 2020). Indeed, *Atox1* silencing reduced breast cell migration *in vitro* (Itoh et al., 2008; Beaino et al., 2014; Kim et al., 2019). Moreover, inhibiting Atox1 delivery to CCS protein by small molecules was effective in killing breast, lung, leukaemia, head, and neck cancer cells *in vitro* and in a mouse xenograft model, while having only minimal effects on healthy cells (Wang et al., 2015).

Here, we provided evidence and expanded on previous results by showing that peptides targeting Atox1 and/or ATP7B MBD3/4 can preferentially elicit cancer cell death when administered in the μM range, owing to an increase in copper concentration within the cell. The presented data in this study proposes that in the presence of copper, the peptides interact with ATP7B and Atox1, which leads to disruption of proper copper trafficking and eventually to an increase in cellular copper concentration.

The use of peptides in drug design studies has become increasingly popular due to their biocompatibility and reduced toxicity, as compared to small molecule inhibitors. Therefore, peptides such as those addressed in the present study may represent an appealing alternative to conventional inhibitory strategies relying on small molecules (Muttenthaler et al., 2021). The designed peptides can interact with both Atox1 and MBD3/4 of ATP7B, although with modest affinity. Remarkably, the peptides triggered a 300–500% increase in cellular copper concentration in MCF7 breast cancer cells without significantly affecting healthy HEK293 cells. In liver cancer cells, an increase in intracellular copper concentration was detected even in the absence of the peptides, as compared to healthy human liver cells. However, the viability of liver cancer cells was also largely affected by the presence of the peptides.

In summary, our results showed how the use of multi-targeting peptides mimicking interactions between copper-trafficking proteins represents a viable strategy for sabotaging copper transport, (i.e., increase cellular copper concentrations) to induce selective cancer cell death. Although our peptides showed lower efficacy and selectivity, as compared to previously identified small molecule inhibitors of Cu trafficking, (Wang et al., 2015), these peptides represent a blueprint for selectively targeting key proteins of the Cu transport route when Cu dys-homeostasis (i.e., elevated cellular Cu(I) concentrations) occurs, such as in metastatic and invasive cancer cells (Blockhuys and Wittung-Stafshede, 2017; Blockhuys et al., 2020). Our results thus advance the possibility and therapeutic relevance of exploiting Cu dys-homeostasis in anti-cancer therapy.

## 4 Experimental section

### 4.1 Model building

A model of the peptide protein adduct was built starting from the representative frame of our previously equilibrated Atox1/MBD4 adducts by taking the portion of Atox1 and MBD4 that best interacted with each other (Qasem et al., 2019).

### 4.2 *In silico* peptide design

Affinity maturation is a Bioluminate tool of Glide used to perform multiple sequential mutations of protein residues in order to optimize binding affinity of the peptide for a target protein (Clark et al., 2019; Negron et al., 2019). Changes in peptide binding affinity due to mutation are calculated from a thermodynamic cycle, which can be represented as:
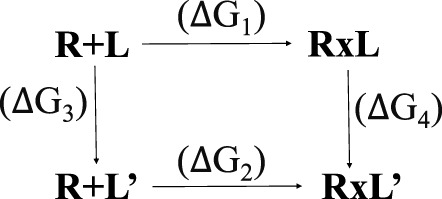


Where R is the receptor, L is the ligand in the parent state, and L′ is the mutated ligand. R + L and R + L′ represent the separated receptor and ligand/mutated ligand. RxL and RxL’ represent the receptor bound to the ligand. A change in binding affinity is described by:

ΔΔG(bind)=ΔG2−ΔG1=ΔG4−ΔG3

The experiment measures ΔG_1_ and ΔG_2_, but actually ΔG_3_ and ΔG_4_ are calculated, to optimize error cancellation in the computational models.

The calculations are carried out with MM-GBSA, which uses an implicit (continuum) solvation model (Chen et al., 2016). In this manner, the Atox1-SMD and MBD4-KER systems were created. All other models (i.e., Atox1-KER, MBD4-SMD, MBD3-SMD, MBD4-KER) were created both by the superimposition method, whereby a structure from the above-mentioned protocol was used as a template, and by molecular docking using the HADDOCK2.2 web server (van Zundert et al., 2016). Regarding MBD3/4, we created two different sets of systems. In the first, the domains were considered as the protein without Cu(I) ions and in the second, in the presence of Cu(I), whereas Atox1 was only considered with Cu(I) ions. The rationale for this lies in the fact that the physiological path of Cu(I) is from Atox1 to MBD3/4.

### 4.3 Classical molecular dynamics

The topology of all systems was created using the Parm14SB AMBER force field (FF) for a protein, (Maier et al., 2015), using the leap module of AmberTools 16 (Case et al., 2005). Parameters for copper atoms and the two cysteine residues coordinating the Cu ion were taken from our previous study (Qasem et al., 2019). Namely, the force constants of the Cu(I) coordination geometry were taken from an earlier report (Op’t Holt and Merz, 2007) and we later refined them in quantum-classical MD simulations (Franco et al., 2014).

For the system involving the MBD3/4 domains, we considered separate models in MD simulations, in contrast to experiments which were performed with a construct in which the two domains are connected by a disordered loop. Nevertheless, we did not expect the local peptide/MBD3/4 interactions to be affected by the other domain and since the two domains are connected by a disordered linker, they freely and randomly move around the simulation box during the MD simulation. This would require us to significantly increase the simulation box size and computational cost of the MD simulation. Therefore, the peptide in complex with Atox1 or with either MBD3 or MBD4 were solvated in a cubic box with the distance between the solute atoms and walls set to 12 Å, filled with TIP3P water molecules, for the methods details see ref (Qasem et al., 2019); Na^+^ ions were added to neutralize the system charge. Bonds involving hydrogen atoms were constrained using the SHAKE algorithm (Qasem et al., 2019). After solvation, all systems contained ∼27,000 atoms.

A time step of 2 fs and a cutoff of 10 Å for non-bonded interactions were used. The systems were carefully heated to a final temperature of 300 K. The temperature control was performed using a Langevin thermostat (Qasem et al., 2019). The box size was equilibrated using the NPT ensemble for 0.5 ns. Pressure control (1 atm) was achieved using a Berendsen barostat (Qasem et al., 2019). Finally, NPT simulations were produced for 200 ns. After careful visual inspection of all trajectories (in some of the systems, SMD or KER dissociated from Atox1/MBD3/4), seven systems were selected for further analysis, namely, Atox1-SMD, MBD3_Cu-SMD, MBD3-KER, MBD3_Cu-KER, and MBD4_Cu-KER obtained from the molecular docking, and MBD4_Cu-KER and MBD4-SMD obtained by manually docking the peptides on the basis of the MBD4/Atox1 adduct obtained in previous simulation studies (Qasem et al., 2019).

### 4.4 Analysis

Hydrogen (H)-bond analysis was performed on the adducts using the *cpptraj* module of AmberTools 16 (the cut-off parameters were 3.3 Å and 35°). The binding free energy (ΔG_b_) was calculated with the MM-GBSA method (Qasem et al., 2019) using the MMPBSA. py program (Qasem et al., 2019) on the equilibrated trajectories, with igb = 8. The peptide-protein adduct was created starting from a representative frame of our previously equilibrated Atox1/MBD4 adducts, taking the portion of Atox1 and MBD4 that best interacted with each other.

### 4.5 Peptide synthesis

Peptides were synthesized using rink-amide resin (GLC, Shanghai, China). The standard Fmoc (9-fluorenylmethoxy-carbonyl)-protected amino acids were coupled with (O-Benzotriazol-1-yl)-N,N,N,N-tetramethyluronium (HBTU, Bio-Lab, Jerusalem, Israel) in N,N-dimethylformamide (DMF, Bio-Lab), combined with N,N-diisopropylethylamine (DIPEA, Merck, Rehovot, Israel) for a 1 h cycle. Fmoc de-protection was achieved with a 20% piperidine/DMF solution (Alfa Aesar, Ward Hill, MA). Ninhydrine tests were performed after each coupling set. Side-chain de-protection and peptide cleavage from the resin were achieved by treating the resin-bound peptides with a 5 ml cocktail of 95% trifluoroacetic acid (TFA, Bio-Lab), 2.5% triisopropylsilane (TIS, Merck), and 2.5% water for 4.5 h. The peptides were washed four times with cold diethyl ether, vortexed, and then centrifuged for 10 min at 3,500 rpm. The peptides were purified by reverse phase HPLC (Luna 5 μm C18; 100 A; 250 mm × 4.6 mm, Phenomenex Torrance, CA). To develop the optimal separation mode, a gradient scouting run was performed by changing the acetonitrile composition from 10 to 90% over 60 min. The column was operated at a flow rate of 2 ml/min, at 120 bar. The masses of the peptides were confirmed by ESI (electrospray ionization) mass spectrometry on a Q-TOF (quadruple time-of-flight) high-resolution 6545 spectrometer (Agilent, Santa Clara, CA) (Supplementary Figures S1, S2 in SI). Peptide samples were typically mixed with two volumes of pre-made dihydrobenzoic acid (DHB) matrix solution, deposited onto stainless steel target surfaces, and allowed to dry at room temperature.

### 4.6 Cloning, expression, and purification of ATP7B MBD3-4

The expression and purification protocol of MBD3-4 was previously described in detail (Pavlin et al., 2019; Qasem et al., 2019; Zaccak et al., 2020). In short, MBD3-4 was cloned into the pTYB12 vector by restriction-free cloning. The clone was expressed in *E. coli* strain Origami 2 grown at 37°C to an optical density of 0.5–0.6 (OD_600_) using terrific broth (TB) medium supplemented with ampicillin and tetracycline as selection factors and induced with 1 mM isopropyl-β-D-thiogalactopyranoside (IPTG) at 18°C overnight. The bacteria were then harvested by centrifugation at 10,000 rpm for 30 min. The pellet was re-suspended in lysis buffer (25 mM Na_2_HPO_4_, pH 8.8, 150 mM NaCl, 20 mM PMSF, 1% Triton X-100) and sonicated (10 min of 30 s pulses at 40% amplitude). Finally, the lysate was centrifuged at 14,500 rpm for 30 min and the supernatant was kept. To purify native ATP7B MBD3-4, the lysate was loaded on a chitin bead column, allowing the ATP7B MBD3/4-intein to bind the resin through its chitin-binding tag. Next, the resin was washed with 50 column volumes of lysis buffer. The intein was self-cleaved using 5 ml dithiothreitol (50 mM DTT) upon incubation for 48 h at 4°C.

Atox1 expression and purification were as for ATP7B, as were described in a series of publications (Levy et al., 2014b; Levy et al., 2016; Levy et al., 2017).

### 4.7 Site-directed spin-labeling (SDSL)

The spin-labeling process was performed in the presence of Cu(I) ions in order to prevent the spin labeling of cysteine residues involved in Cu(I) binding. Before labeling, 10 mM DTT was added to the protein solution and mixed overnight (o.n.) at 4°C. DTT was dialyzed away using 1 kDa dialysis cassettes (Pierce). Next, 0.25 mg of S-(2,2,5,5-tetramethyl-2,5-dihydro-1H-pyrrol-3-yl) methyl methanesulfonothioate (MTSSL, TRC) was dissolved in 15 μL dimethyl sulfoxide (DMSO, Bio lab) and added to 0.75 ml of a 0.01 mM protein solution (20-fold molar excess of MTSSL). The protein solution (with Cu(I)) was then vortexed overnight at 4°C. Free spin label and Cu(I) ions were removed by several dialysis cycles over 4 days. The removal of Cu(I) was verified using 0.1 mM KCN solution. The mass of the spin-labeled protein was confirmed by mass spectrometry, and concentration was determined by a BCA assay.

### 4.8 Cu(I) addition

For EPR measurements: Cu(I) (tetrakis (acetonitrile) copper(I) hexafluorophosphate) was added to the protein solution under nitrogen gas to preserve anaerobic conditions. No Cu(II) EPR signal was observed at any time.

### 4.9 X-band continuous-wave electron paramagnetic resonance experiments

CW-EPR spectra were recorded using an E500 Elexsys Bruker spectrometer operating at 9.0–9.5 GHz, equipped with a super-high-sensitivity CW resonator. The spectra were recorded at room temperature (292 ± 5 K), at a microwave power of 20.0 mW, a modulation amplitude of 1.0 G, a time constant of 60 ms, and a receiver gain of 60.0 dB. Samples were measured in 1.0 mm quartz tubes (Wilmad-LabGlass, Vineland, NJ). The final spin-labeled protein concentration was between 0.01 and 0.03 mM.

### 4.10 Q-band double electron electron resonance (DEER) experiments

DEER experiments (π/2 (ν_obs_)—τ1—π(ν_obs_)—t′—π(ν_pump_)—(τ1+τ2—t′)—π(ν_obs_)—τ2—echo) were carried out at 50 ± 1.0 K on a Q-band Elexsys E580 spectrometer (equipped with a 2 mm probe head and AmpQ TWT). A two-step phase cycle was employed on the first pulse. The echo was measured as a function of t′, whereas τ2 was kept constant to eliminate relaxation effects. The durations of the observer π/2 and π pulses were 12 and 24 ns, respectively. The duration of the π pump pulse was 24 ns, and the dwell time was 16 ns. τ1 was set to 200 ns. The observer frequency was 33.8 GHz, the pump frequency was 33.86 GHz and the magnetic field was 12025 G. Samples were measured in 1.6 mm capillary quartz tubes (Wilmad-LabGlass). The data were analyzed using the DeerAnalysis 2019 program (Deeranalysis et al., 2006).

### 4.11 Circular dichroism characterization

Circular dichroism CD measurements were performed using a Chirascan spectrometer (Applied Photophysics, United Kingdom) at room temperature. Measurements were carried out in a 1 mm optical path length cell. Data were recorded from 190 to 260 nm with a step size and a bandwidth of 1 nm. Spectra were obtained after background subtraction. CD measurements were conducted on ATP7B MBD3/4, Atox1, and peptides after dialysis with water.

### 4.12 Fluorescence labelling

The labelling was performed with reactive dyes using N-hydroxysuccinimide (NHS)–ester chemistry. The protein concentration was adjusted to 20 μM using HEPES buffer. Briefly, the solid fluorescent dye was dissolved in DMSO at a concentration of about 1.3 mM and mixed thoroughly. Before mixing the protein and dye, the concentration of the dye was adjusted to twice that of the protein using HEPES buffer. Then, the protein and fluorescent dye solutions were mixed at a 1:1 ratio and incubated for 30 min at room temperature in the dark. Unreacted “free” dye was eliminated by gel filtration (Sephadex G25, GE Healthcare). Purity was monitored by measuring the ratio of protein to dye (spectroscopically, for example, by measuring absorption at 280 nm for protein and 650 nm for the dye; molar absorbance: 250,000 M^−1^ cm^−1^) after the clean-up procedure.

### 4.13 Microscale thermophoresis (MST) assay

MBD3-4 and Atox1 were labeled on lysine residues with red fluorescent NT-647-NHS using the NanoTemper labeling kit. Measurements were carried out in 25 mM Na_2_HPO_4_, pH 8, 150 mM NaCl, and 0.05% Tween 20 buffer solution. PCR microtubes were prepared with the peptides and target protein solutions. Peptide concentrations ranged from 122 μM to 4 mM, whereas protein concentrations remained constant at 50 nM. After incubation for 30 min at room temperature, the samples were loaded into standard capillaries, and measurements were performed using 20, 40, and 60% MST power and 20% LED power at 25°C on a Monolith NT.115 instrument. Normalized fluorescence was detected at a ratio of [F_norm_ = F_hot_/F_initial_]. Fluorescence was measured before IR-laser heating (F_initial_) and after a specific IR-laser heating (F_hot_) time. Kd values were obtained after fitting the experimental data with the following equation:

$$F_{norm}=\frac{\left(\left[protein\right]+\left[peptide\right]+K_{d}\right)-\sqrt{{\left(\left[protein\right]+\left[peptide\right]+K_{d}\right)}^{2}-4∙\left[protein\right]\left[peptide\right]}}{2\left[protein\right]}$$

### 4.14 Cell culture and treatment

HEK293 and MCF7 cells were cultured in Dulbecco’s modified Eagle’s medium (DMEM) high glucose medium, with fetal bovine serum (10%), L-glutamine (1%), and PSNS antibiotic (1%). The HepG2 cell line was cultured in Eagle’s minimum essential medium (EMEM), with fetal bovine serum (10%), L-glutamine (1%) and PSNS antibiotic (1%). The THLE-3 cell line was cultured in BGEM medium according to manufacturer’s instructions. All cell line types were cultured at 37°C with 5% CO_2_. The culture media was changed every 2 days. At 80% confluence, the cells were harvested using 0.25% trypsin and sub-cultured into 75 cm^2^ flasks, 24-well plates, or 96-well plates, according to the experiment. The cells were then treated with a range of peptide concentrations suspended in medium without serum for 24 h. After the 24 h treatment, the various toxicity end points were evaluated in the control and peptide-exposed cells.

### 4.15 Cell toxicity (MTT assay)

Peptides were introduced at various concentrations (three triplicates per concentration) ranging from 0 to 1 mM and incubated with the different cell lines (HEK293/MCF7/HepG2/THLE-3) for 24 h at 37°C. The medium was removed and 0.1% of 3-(4,5-dimethylthiazol-2-yl)-2,5-diphenyltetrazolium bromide in PBS buffer was added and the cells were incubated for 2 h at 37°C. The PBS buffer was collected from the plates and 200 µl of DMSO were added and incubated with shaking for 30 min at room temperature. Lysates were transferred to 96-well plates and absorption values were collected using a Synergy plate reader at 570 nm wavelength. Calculations were based on the average of three repetitions. The absorption of the control (without peptide) was determined at 100%; all peptide concentrations were compared to the control sample. The MTT assay assesses cell viability by measuring the enzymatic reduction of yellow tetrazolium MTT to a purple formazan.

### 4.16 ^64^Cu(II) cell experiments

The HEK293/MCF7/HepG2/THLE-3 cell lines were grown in 6-well plates until 50% confluence, as described above. Then, 600 µl of SMD or KER peptide was added to relevant wells. The cells were grown for 24 h at 37°C and 5 µCi of ^64^Cu(II) were added to each wall. At chosen time intervals, the medium was removed, the cells were washed with 4 ml PBS, 600 µl of RIPA buffer was added and the cells were scraped. The removed cells were measured for radioactivity using a gamma counter. All measurements were performed after fixing the efficiency detector for 100%. The sensitivity of the gamma counter (2480 PerkinElmer) is 0.02 ± 0.005% relative activity uptake.

### 4.17 Western blot experiments

The cell lines cultures used for testing were grown in cell culture flasks and incubated at 37°C with 5% CO_2_ for a few days. After incubation, the culture was washed with cold PBS and lysed by adding RIPA Buffer. The samples containing 50 μg of total protein from each cell line were separated by SDS-PAGE and transferred to a nitrocellulose membrane using a transfer apparatus according to the manufacturer’s protocols (Bio-Rad). After incubation with 3% milk solution in TBST (10 mM Tris, pH = 8.0, 150 mM NaCl, 0.5% Tween 20) for 60 min, the membrane was incubated with antibodies against human Ctr1 (GeneTex SLC314, 1: 1000) or Actin DSHB JLA20, 1: 500) at 4°C over night. The membrane was washed with TBST three times for 10 min and incubated with a 1: 20000 dilutions of peroxidase-conjugated secondary antibody for 60 min. The blot was washed with TBST three times for 10 min and developed with the ECL system (Bio-Rad) according to the manufacturer’s protocols.

## Data Availability

The original contributions presented in the study are included in the article/Supplementary Material, further inquiries can be directed to the corresponding authors.
