# Supplementary material for: Disrupting Cu trafficking as a potential therapy for cancer
Source: Front Mol Biosci. 2022 Oct 10;9:1011294. doi: 10.3389/fmolb.2022.1011294 (PMC9589254; doi:10.3389/fmolb.2022.1011294)
Supplement: Supplementary file 1 [file DataSheet1.PDF]

Disrupting Cu Trafficking as a potential therapy for cancer

Zena Qasem[1,#], Matic Pavlin[2,3,#], Ida Ritacco[2,4], Matan Y. Avivi[5], Shelly Meron[1], Melanie Hirsch[1], Yulia Shenberger[1], Lada Gevorkyan-Airapetov[1], Alessandra Magistrato[2,*], Sharon Ruthstein[1,*]

[1] Department of Chemistry and the Institute of Nanotechnology and advanced Materials (BINA), Bar-Ilan University, Ramat-Gan 52900, Israel.

[2] Department National Research Council of Italy (CNR) - Institute of Material (IOM) c/o International School for Advanced Studies (SISSA), via Bonomea 265, 34136 Trieste, Italy.

[3] Department of Catalysis and Chemical Reaction Engineering, National Institute of Chemistry, Hajdrihova 19, 1001 Ljubljana, Slovenia.
[4] Department of Chemistry, University of Salerno, Italy.

[5] The Mina & Everard Goodman Faculty of Life-Sciences, Bar-Ilan University, Ramat-Gan 52900, Israel.

# Equal Contribution

*** Correspondence:**
Sharon Ruthstein, Sharon.ruthstein@biu.ac.il; Alessandra Magistrato, alema@sissa.it

Supporting Information

Table of Contents	Pages
HPLC and Mass Spec data (Figs. S1-S2)	2-3
CW EPR Experiments (Fig. S3)	4
CD data (Fig. S4)	5
Additional MST experiments (Fig. S5-S6)	6
Additional cell experiments (Fig. S7)	7
Western blot of Ctr1	7
Supplementary tables (Tables S1-S5)	8-14

ESI-MS

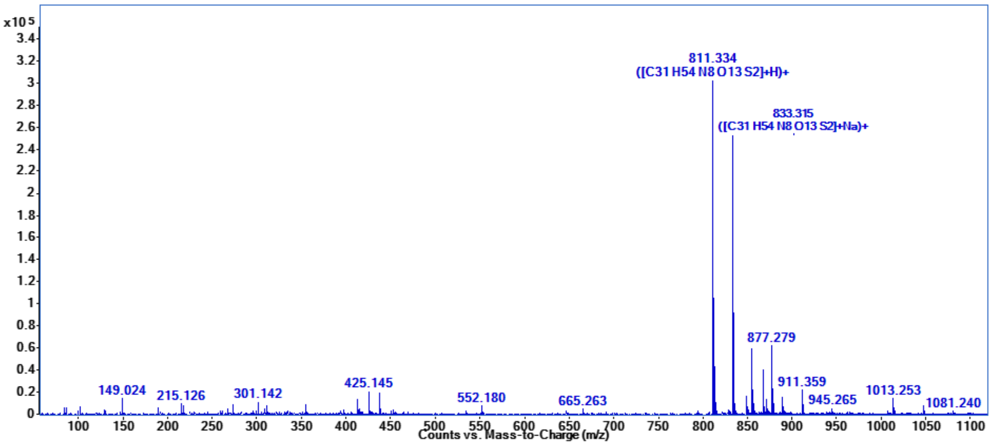

MS Spectrum Peak List

m/z	Calc *m/z*	Diff(ppm)	z	Abund	Formula	Ion
811.334	811.332	-1.3	1	119808.8	C31H54N8O13S2	(M+H)+
812.336	812.335	-1.22	1	41551.46	C31H54N8O13S2	(M+H)+
813.334	813.333	-1.3	1	17135.42	C31H54N8O13S2	(M+H)+
814.335	814.334	-0.58	1	4490.13	C31H54N8O13S2	(M+H)+
833.315	833.314	-1.21	1	65941.09	C31H54N8O13S2	(M+Na)+
834.318	834.317	-0.9	1	23808.52	C31H54N8O13S2	(M+Na)+
835.315	835.315	0.77	1	10407.3	C31H54N8O13S2	(M+Na)+
836.314	836.316	2.91	1	2888.12	C31H54N8O13S2	(M+Na)+

HPLC

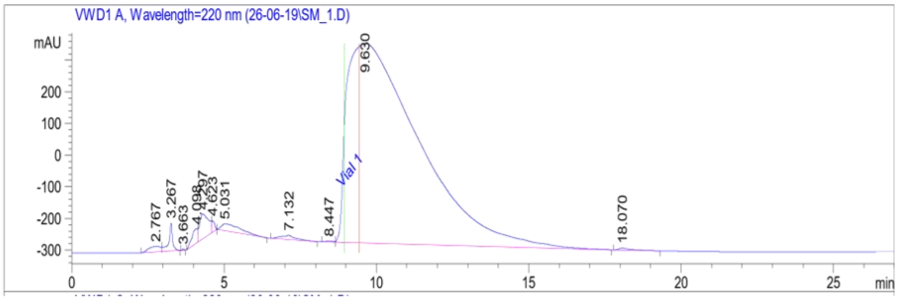

Peak #	RetTime [min]	Type	Width [min]	Area [mAU*s]	Height [mAU]	Area %
1	2.767	BV	0.3632	477.63174	17.73596	0.4353
2	3.267	VB	0.1199	776.17584	88.43222	0.7073
3	3.663	BB	0.0739	19.69630	4.29987	0.0179
4	4.098	BV	0.1708	598.27136	45.37130	0.5452
5	4.297	VV	0.2662	1569.02832	79.00199	1.4298
6	4.623	VB	0.0815	232.30740	37.12670	0.2117
7	5.031	BB	0.6336	1008.23962	21.36476	0.9188
8	7.132	BB	0.4087	437.88052	13.65303	0.3990
9	8.447	BV	0.2436	54.06602	3.30018	0.0493
10	9.630	VB	2.0941	1.04408e5	631.44116	95.1463
11	18.070	BB	0.3665	152.89111	5.90692	0.1393
Totals :				1.09734e5	947.63410	

Figure S1: Characterization of the SMD peptide by high resolution + ESI MS and HPLC.

ESI-MS

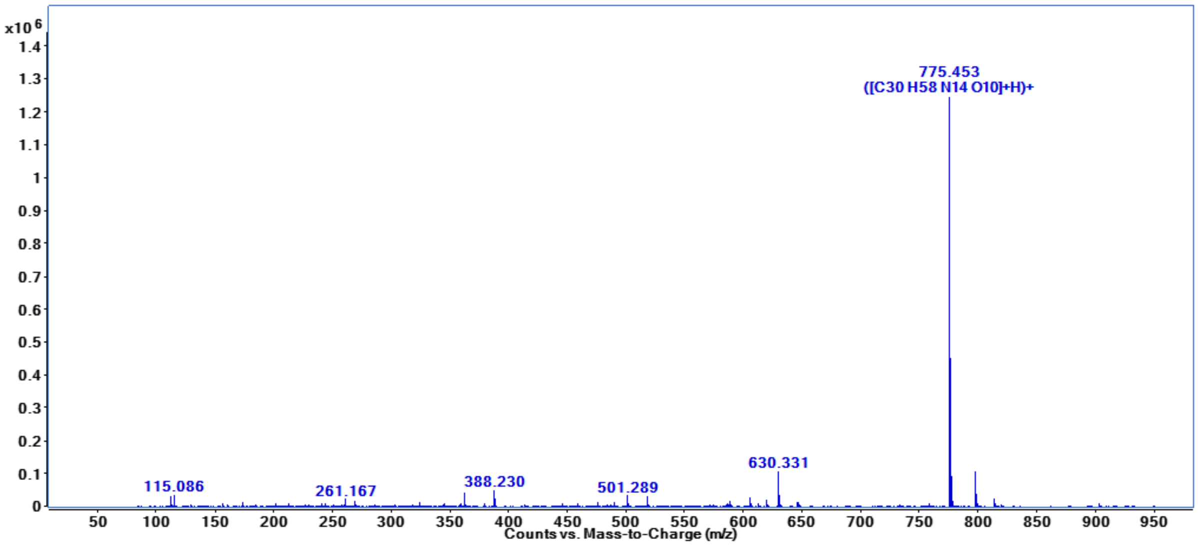

MS Spectrum Peak List

m/z	Calc *m/z*	Diff(ppm)	z	Abund	Formula	Ion
775.453	775.453	-0.05	1	95666.41	C30H58N14O10	(M+H)+
776.456	776.456	0.39	1	34749	C30H58N14O10	(M+H)+
777.457	777.458	1.11	1	6950.07	C30H58N14O10	(M+H)+
778.46	778.461	1.33	1	1373.27	C30H58N14O10	(M+H)+
779.46	779.463	3.4	1	127.55	C30H58N14O10	(M+H)+
797.433	797.435	3.13	1	1467.67	C30H58N14O10	(M+Na)+
798.436	798.438	2.76	1	474.28	C30H58N14O10	(M+Na)+
799.431	799.44	11.36	1	175.56	C30H58N14O10	(M+Na)+

HPLC

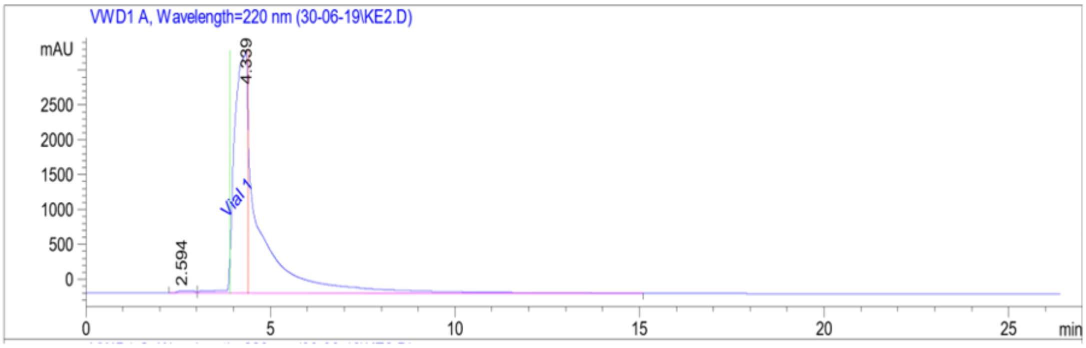

Peak #	RetTime [min]	Type	Width [min]	Area [mAU*s]	Height [mAU]	Area %
1	2.594	BV	0.3737	874.32672	30.98218	0.5580
2	4.339	VB	0.5336	1.55810e5	3488.62476	99.4420
Totals :				1.56685e5	3519.60693	

Figure S2: Characterization of the KER peptide by high resolution + ESI MS and HPLC.

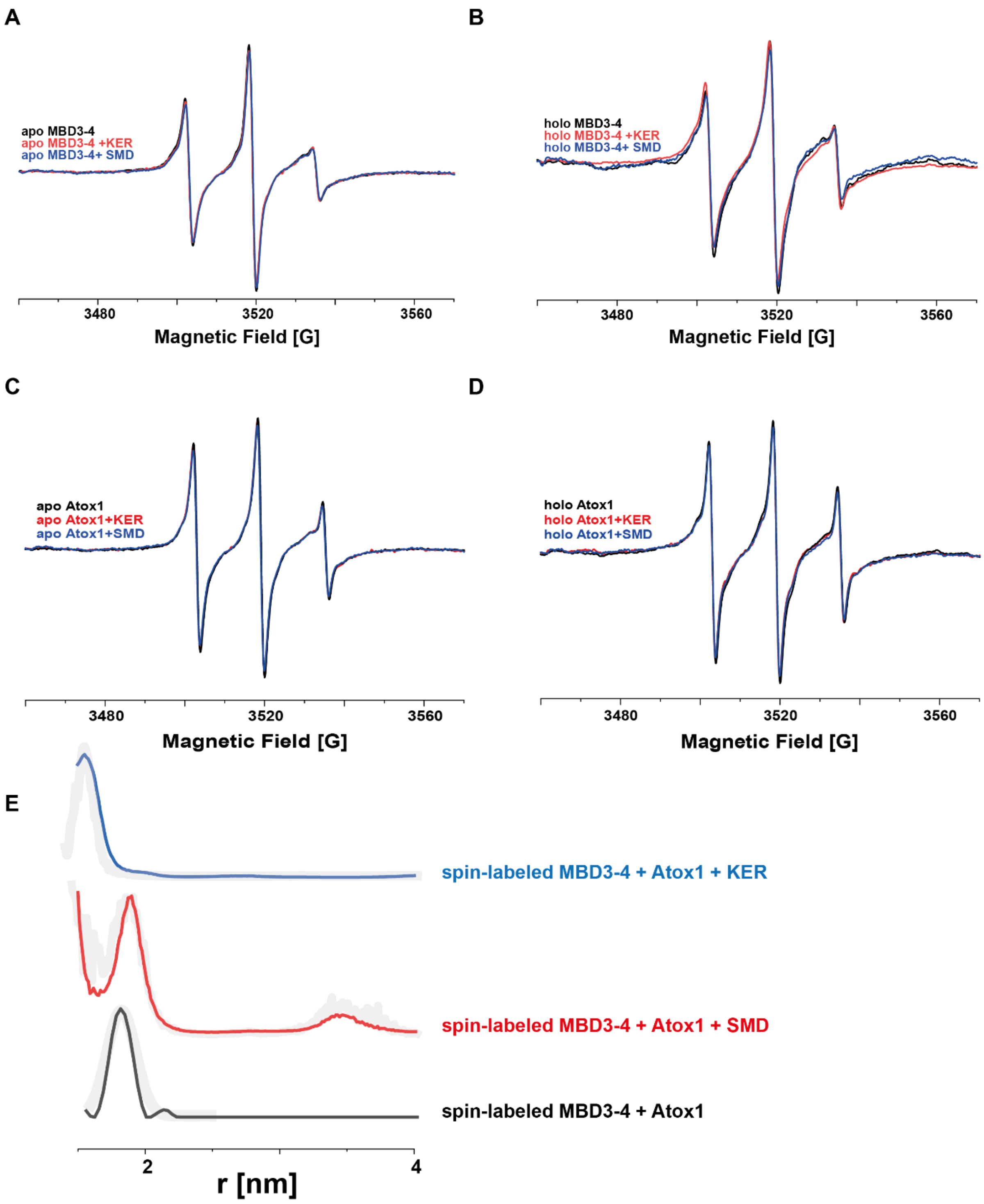

Figure S3: CW-EPR spectra in the presence of the SMD and KER peptides of (**A**) spin-labeled apo MBD3-4; (**B**) spin-labeled holo MBD3-4; (**C**) spin-labeled apo Atox1; and (**D**) spin-labeled holo Atox1. (**E**) Q-band distance distribution functions obtained using the DEER experiments and DeerNET analysis for spin-labeled MBD3-4 in the presence of Atox1 and KER/SMD peptides.

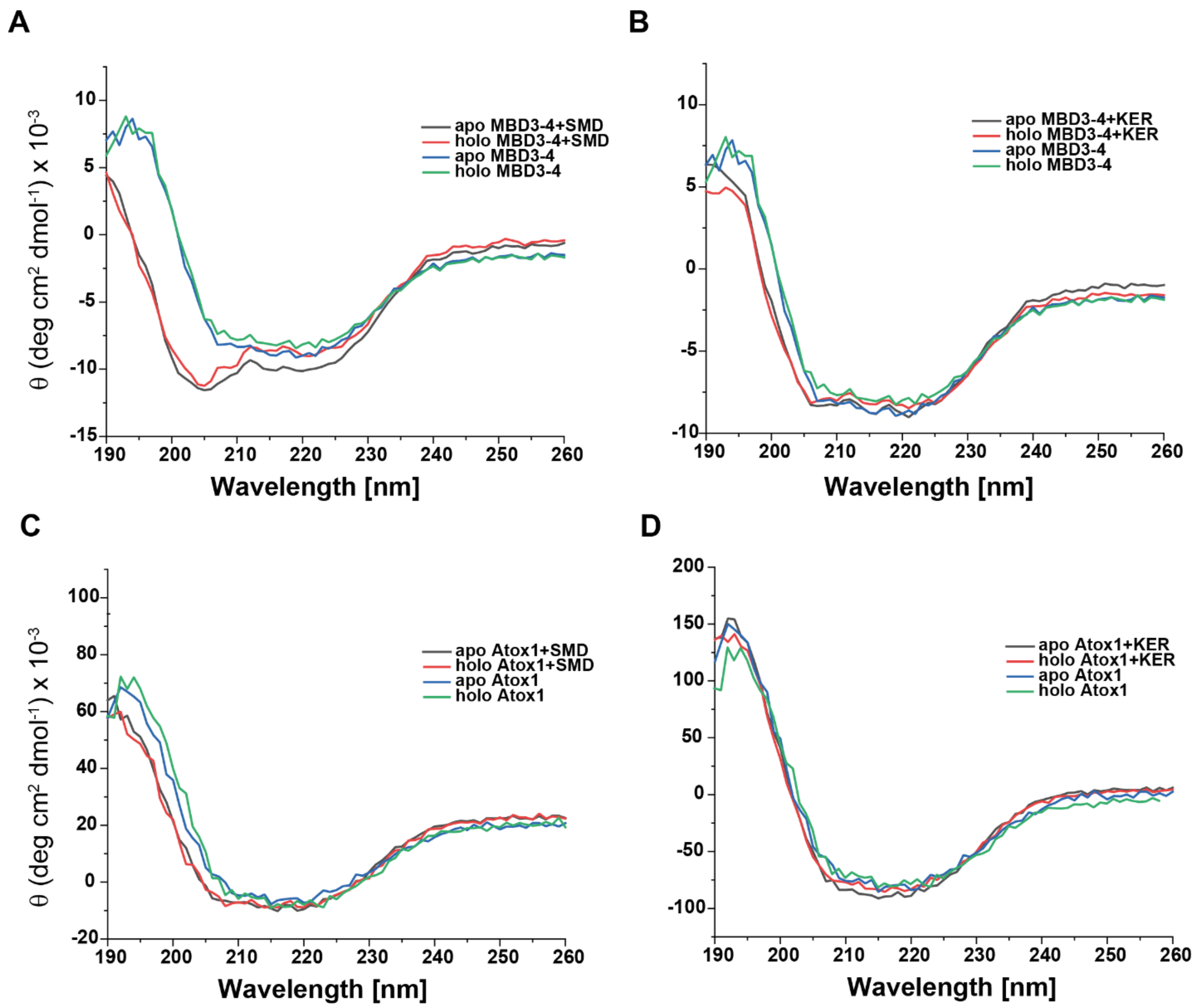

Figure S4: CD spectra in the presence and absence of Cu(I) for MBD3-4 with SMD and KER peptide (**A**) and (**B**), respectively; Atox1 with SMD and KER peptide (**C**) and (**D**), respectively.

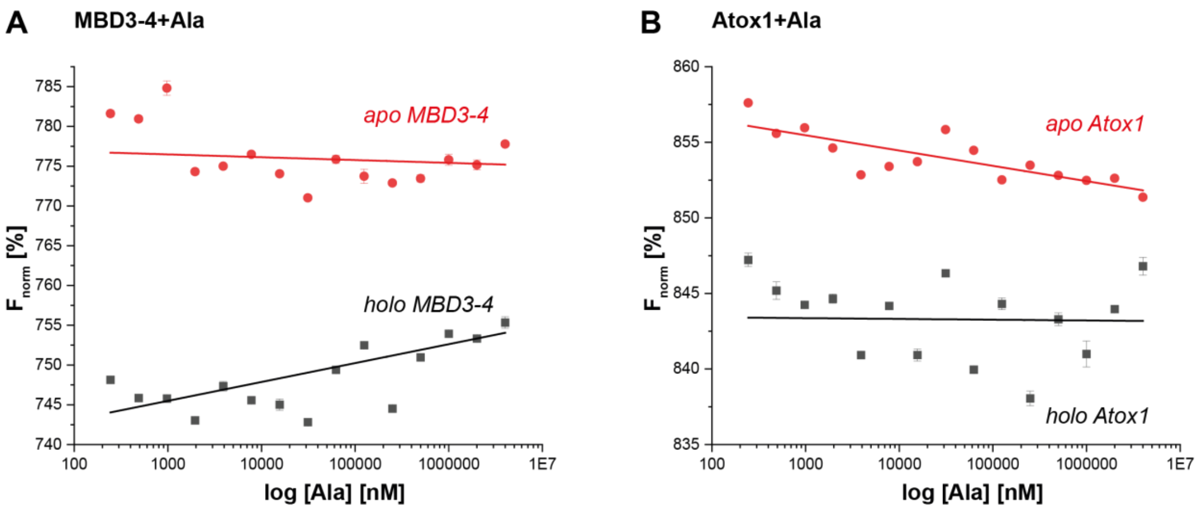

Figure S5: Binding curves generated by MST binding of Ala peptide (GAGAAA) for (**A**) MBD3-4 protein and (**B**) Atox1 protein, in the presence (the black curves) and absence (the red curves) of Cu(I).

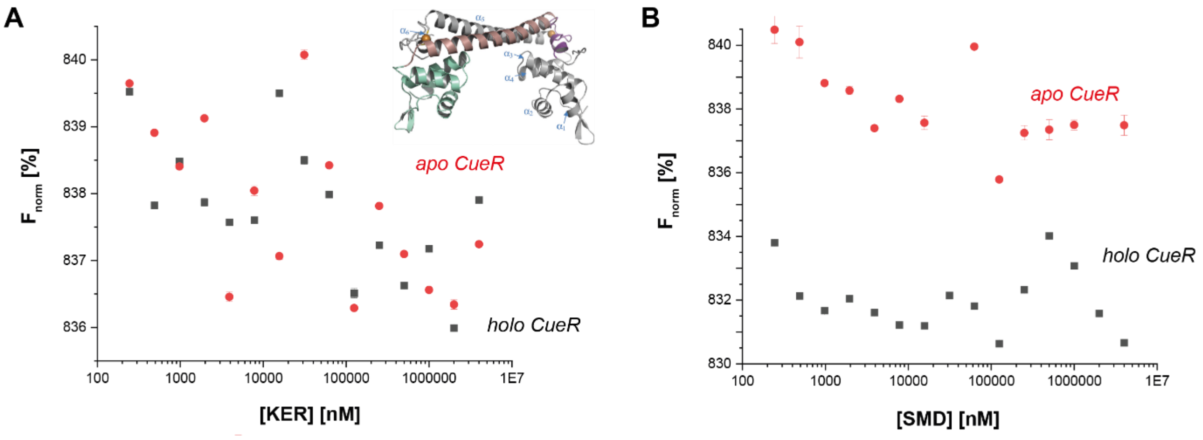

Figure S6: Binding curves generated by MST binding of CueR protein (PDB 1Q05) for (**A**) KER peptide and (**B**) SMD peptide, in the presence (the black curves) and absence (the red curves) of Cu(I).

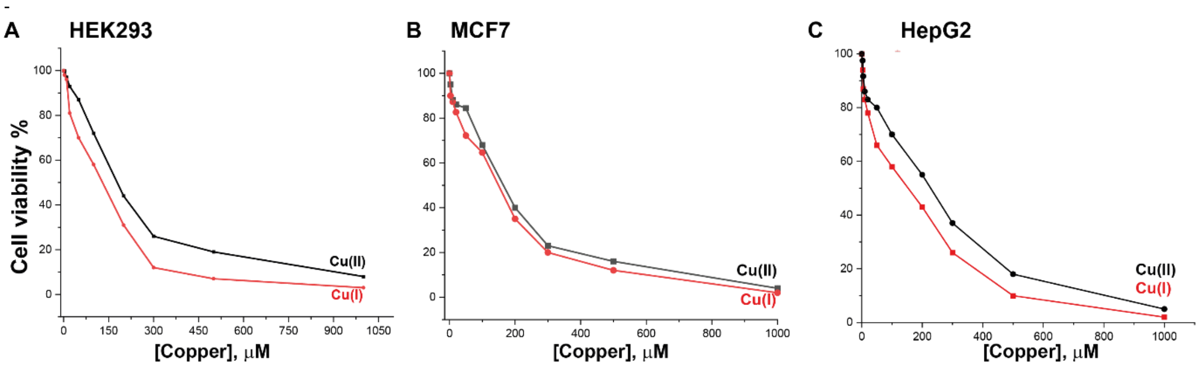

Figure S7: Cell viability experiments as a function of Cu(II) and Cu(I) concentration in HEK293 cells (**A**), MCF7 cells (**B**), and HepG2 cell (**C**).

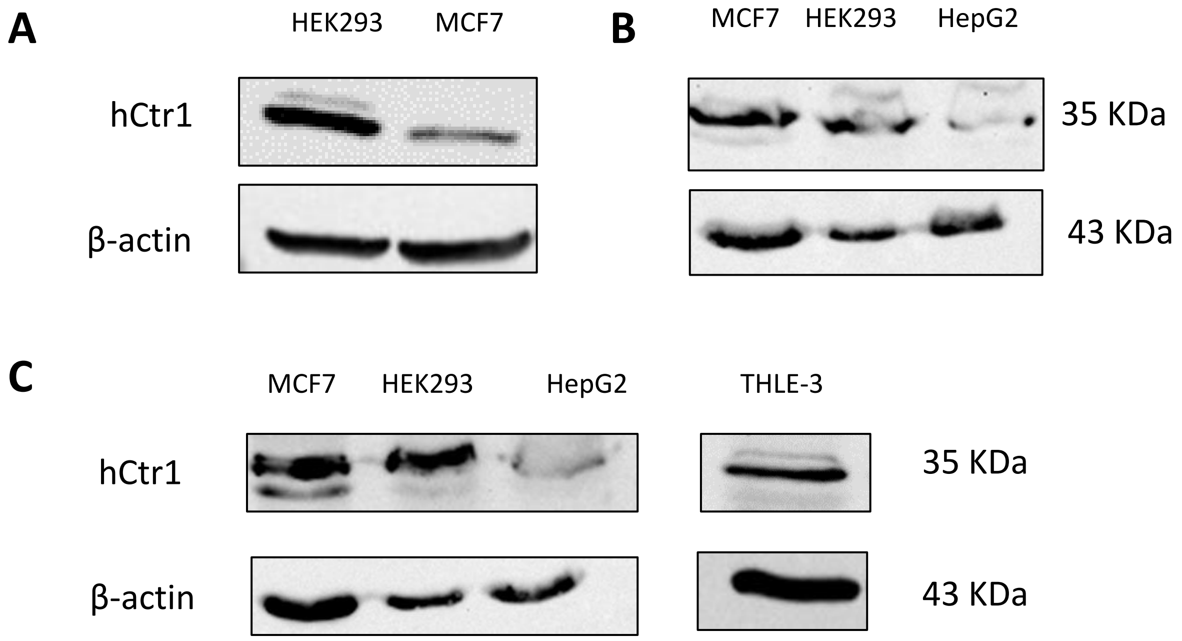

Figure S8: Expression of copper transporter, Ctr1, in several cell lines by western blot. Actin was used as a loading control. (**A**), **(B)** and **(C)** present different experiments.

Table S1. Summary of the docking and molecular dynamics (MD) simulations performed on all apo and holo KER or SMD in complex with Atox1, MBD3, and MBD4. MD simulations started from the pose obtained after peptide optimization simulations and after direct docking simulation. When no relevant docking pose was found, the structure is not shown. The structure of the highest populated cluster as obtained from 200 ns long-MD simulations is shown. Peptide/target structures observed to be stable after 200 ns long MD simulations are reported and highlighted with green squares and are discussed in the main text of the manuscript.

System	Pose after peptide optimization	Docking of the peptide
MBD4-KER	No relevant binding pose after MD 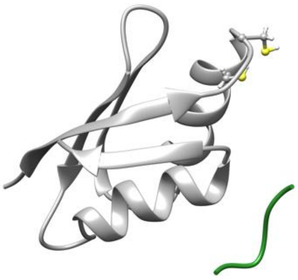	No relevant binding pose after MD from different docking starting poses
MBD4-KER-Cu	Stable binding pose 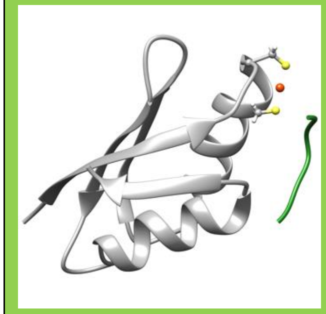	Stable binding pose 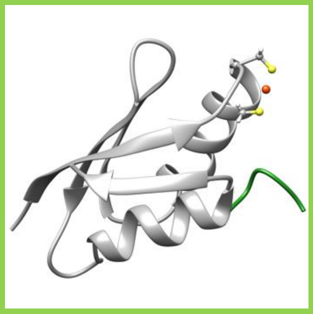
MBD4-SMD	Stable binding pose 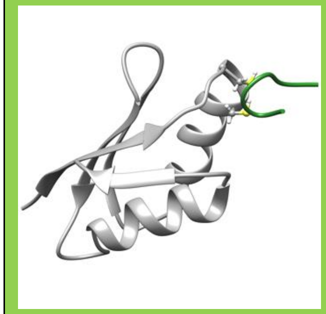	SMD dissociated in MD
MBD4-SMD-Cu	No relevant binding pose after MD 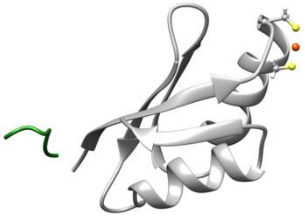	No stable binding pose after MD starting from different docking poses

MBD3-KER	No relevant binding pose after MD<br>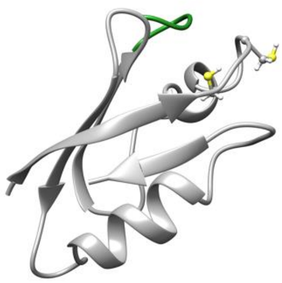	Stable binding pose<br>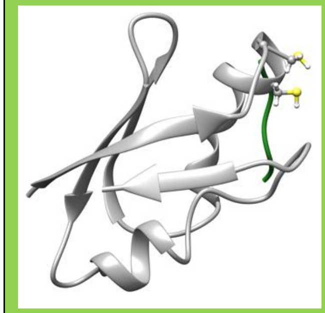
MBD3-KER-Cu	KER dissociatied in MD run	Stable binding pose<br>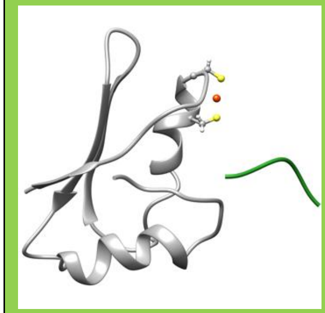
MBD3-SMD	No relevant binding pose after MD<br>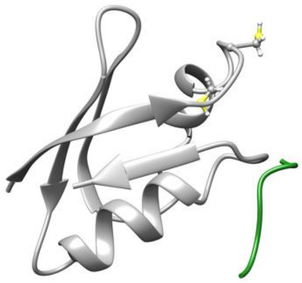	No stable binding pose after MD starting from different docking poses
MBD3-SMD-Cu	No relevant binding pose after MD<br>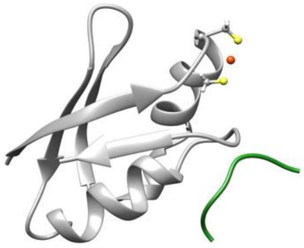	Stable binding pose<br>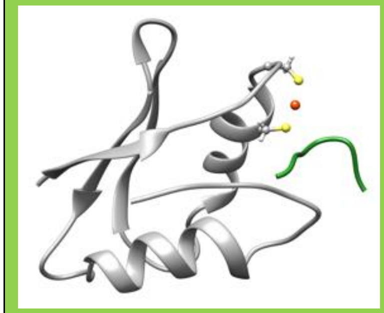

Atox-SMD-Cu	No relevant binding pose after MD 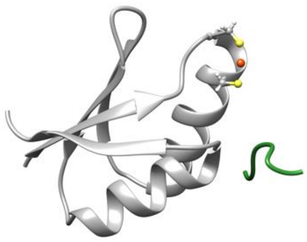	Stable binding pose 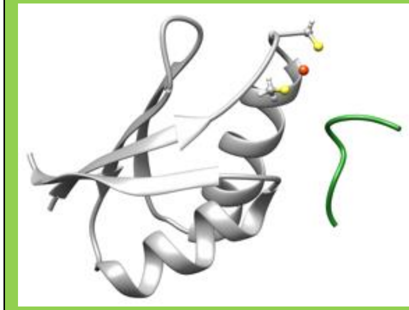
Atox-KER-Cu	No relevant binding pose after MD 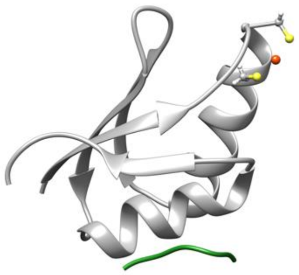	No relevant binding pose from docking

Table S2. Hydrogen bond persistency (%) between the SMD and KER peptide and their target Atox1 and the metal binding domain MBD 3 and 4 of ATP7B protein in holo or apo form. When the same moiety is involved during the simulations in hydrogen bonds with different atoms of the same group sum of single contributions is reported. Persistence above 50, in between 20 and 50, and below 20 % are reported in green, yellow, and black, respectively.

**Holo Atox1**	**SMD**	**Persistence (%)**
Arg21@NH1-HH1	Met4@O	17.3
Arg21@NH1/2-HH*<br>Arg21@NH1/2-HH*<br>Arg21@NE-HE	Glu7@OE1/2<br>Glu7@O<br>Glu7@O	8.1 + 8.0 + 7.3 + 6.9<br>7.3<br>6.8
Lys57@O	Glu7@N-H	9.0
Thr58@O	Ser1@OG-HG	4.6
Cu	Asp3@OD1/2	METAL COORD.
**Apo MBD4**	**SMD**	**Persistence (%)**
Thr369@N-H	Asp3@O	61.9
Cys370@N-H	Asp3@O	50.3
**Holo MBD3**	**SMD**	**Persistence (%)**
His267@N-H	Glu7@OE1	26.1
Cys268@N-H	Glu7@OE1	3.5
Ser270@OG-HG	Asp3@OD1/2	10.4 + 9.9
Cys271@O	Ser1@N-H1/2/3<br>Ser1@OG-HG	6.0 + 5.6 + 5.5<br>2.3
Cys271@SG	Asp3@N-H	33.0
Cys271@SG	Met4@N-H	28.7
Asn274@ND2-HD21<br>Asn274@OD1	Ser1@O<br>Ser1@OG-HG	39.0<br>5.7
Asn274@ND2-HD21	Asp3@OD1/2	3.6 + 2.4
Asn278@OD1	Ser1@OG-HG	5.0
Ile315@O	Ser1@OG-HG	6.9
Pro320@O	Ser1@N-H1/2/3	3.1 + 3.0 + 2.8
Pro320@O	Met2@N-H	51.5
Asn322@O	Ser1@N-H1/2/3	6.0 + 5.5 + 5.2

**Apo MBD3**	**KER**	**Persistence (%)**
Ser270@OG-HG<br>Ser270@OG	Arg6@O/OXT<br>Arg6@N-H	8.1 + 7.5<br>2.8
Cys271@SG-HG	Glu2@OE1/2	1.1
Asn274@ND2-HD21	Glu2@OE1/2	3.2
Asn274@ND2-HD*<br>Asn274@O<br>Asn274@OD1	Arg3@O<br>Arg3@NH1/2-HH*<br>Arg3@NH1/2-HH*<br>Arg3@NE-HE	11.3<br>13.7 + 2.3<br>3.3<br>1.8
Asn274@ND2-HD2<br>Asn274@OD1	Thr4@O<br>Thr4@N-H	23.5<br>2.9
Asn274@OD1	Ser5@N-H<br>Ser5@OG-HG	5.5<br>1.0
Asn274@OD1	Arg6@NH1/2-HH*<br>Arg6@N-H<br>Arg6@NE-HE	9.0<br>8.1<br>7.5
Glu276@OE1/2	Arg6@NH1/2-HH*	16.3 + 12.4 + 8.4 + 6.1 + 1.1
Glu277@OE1/2	Lys1@NZ-HZ1/2/3	1.3 + 1.3 + 1.2 + 1.2
Glu277@OE1/2	Arg3@NH1/2-HH*<br>Arg3@NE-HE	2.1 + 1.6 + 1.1 + 1.1<br>2.2 + 1.7
Glu277@OE1/2	Thr4@OG1-HG1<br>Thr4@N-H	10.8 + 9.0<br>9.1 + 7.1
Glu277@O	Ser5@OG-HG	2.9
Glu277@OE1/2	Arg6@NH1/2-HH*<br>Arg@NE-HE	12.5 + 9.7 + 3.5 + 2.5 + 1.5 + 1.0<br>7.6 + 5.2
Asn278@OD1	Arg3@NH1/2-HH*	5.6 + 1.7

Asn278@ND2-HD*	Thr4@O	9.3
Asn278@OD1	Arg6@NE-HE<br>Arg6@N-H	8.4<br>4.4
Gly280@O	Lys1@N-H1/2/3	1.4
Gln281@NE2-HE*	Lys1@O	1.2
Gln281@OE1	Glu2@N-H	1.9
Gln281@OE1<br>Gln281@NE2-HE*	Arg6@N-H<br>Arg6@O/OXT	7.6<br>1.9 + 1.7
Glu316@OE1/2	Arg3@NH1/2-HH*	40.1 + 33.1 + 30.0 + 25.2
Glu316@OE1/2	Arg6@NH1/2-HH*	9.2 + 7.9 + 7.5 + 7.2 + 1.4
Leu318@O	Arg3@NH1/2-HH*	4.0
**Holo MBD3**	**KER**	**Persistence (%)**
Ser270@OG	Arg3@NH1/2-HH*	2.5
Cys271@O	Lys1@N-H*	6.9 + 6.0 + 5.7
Cys271@SG	Arg3@NH1/2-HH*<br>Arg3@NE-HE	47.7 + 7.2 + 6.4<br>26.9
Asn274@O<br>Asn274@OD1	Lys1@NZ-HZ*<br>Lys1@NZ-HZ*	6.5 + 6.3 + 6.2<br>5.4 + 5.1 + 4.8
Glu277@OE1/2	Lys1@NZ-HZ*	7.6 + 7.4 + 7.1 + 3.4 + 3.3 + 2.5
Glu277@OE1/2	Arg3@NH1/2-HH*	19.1 + 18.1 + 1.9 + 1.6
Asn278@OD1	Lys1@NZ-HZ*	18.2 + 17.8 + 16.8
Pro320@O	Glu2@N-H	86.7
Asn322@OD1	Lys1@NZ-HZ*	3.1 + 3.0 + 2.6
**Holo MBD4**	**KER (pose 1)**	**Persistence (%)**
Cys373@SG	Arg3@NH1/2-HH*<br>Arg3@NE-HE	75.1 + 71.5 + 8.9<br>4.3
Gln383@NE2-HE2*	Thr4@O	13.4 + 7.1
Gln383@OE1	Ser5@OG-HG	5.8
Gln383@NE2-HE2*<br>Gln383@OE1<br>Gln383@O	Arg6@O/OXT<br>Arg6@N-H<br>Arg6@NH1/2-HH*	1.2<br>18.1<br>1.3
Glu385@OE1/2	Arg6@NH1/2-HH*<br>Arg6@NE-HE	15.1 + 14.7 + 14.4 + 14.3 + 2.1 + 1.4<br>1.8 + 1.4
Glu412@OE1/2	Arg6@NH1/2-HH*	15.0 + 13.1 + 8.3 + 6.6
Asp419@OD1/2	Arg6@NH1/2-HH*<br>Arg6@NE-HE	23.2 + 7.1 + 4.0 + 3.4<br>28.5 + 4.9
Met420@O	Arg3@NH1/2-HH*	4.6
**Holo MBD4**	**KER (pose 2)**	**Persistence (%)**
Thr369@OG1-HG1<br>Thr369@N-H	Glu2@OE1/2<br>Glu2@OE1/2	12.7 + 10.9<br>1.6 + 1.5
Cys373@SG	Lys1@NZ-HZ1/2/3	7.2 + 5.7 + 5.1
Cys373@SG	Arg3@NH1/2-HH*<br>Arg3@NE-HE	92.1<br>24.9
Ser376@OG-HG<br>Ser376@OG<br><br>Ser376@O	Arg6@O/OXT<br>Arg6@NE-HE<br>Arg6@NH1/2-HH*<br>Arg6@NH1/2-HH*	46.2 + 1.7<br>6.5<br>1.7<br>2.8
Asp419@OD1/2<br>Asp419@OD1/2<br>Asp419@O	Arg6@NH1/2-HH*<br>Arg6@NE-HE<br>Arg6@NH1/2-HH*	12.0 + 12.0 + 11.6 + 8.3 + 2.6 + 2.2 + 1.3 + 0.8<br>1.7<br>1.3 + 1.0
Met420@O	Arg3@NH1/2-HH*	53.7

Table S3. Binding free energy (kcal/mol) per residue calculated for the SMD and KER peptides and their target protein with the Molecular Mechanics/Generalized Born Surface Area (MM_GBSA) program.

**Holo Atox1/SMD**			
Atox1 residues ΔGb		SMD residues ΔGb	
Cys12	2.6 ± 1.0	Asp3	-1.5 ± 1.5
Gly14	-1.5 ± 0.8	Met4	-3.4 ± 1.5
Cys15	3.3 ± 1.2	Ile6	-4.7 ± 1.6
Ala18	-2.1 ± 0.6	Glu7	-1.6 ± 1.5
Arg21	-2.7 ± 1.9		
Val22	-1.0 ± 0.5		
Lys25	-1.0 ± 1.3		
Lys57	-1.0 ± 1.3		
Lys60	-1.1 ± 1.4		

**Holo MBD3/SMD**			
MBD3 residues ΔGb		SMD residues ΔGb	
Cys268	2.1 ± 2.7	Ser1	10.7 ± 3.2
Asn274	-2.1 ± 1.2	Met2	-4.1 ± 1.9
Pro320	-4.2 ± 1.1	Asp3	-2.4 ± 2.9
Asn322	-1.1 ± 1.1	Met4	-2.6 ± 1.8
Phe323	-1.2 ± 0.7	Ser5	-1.9 ± 2.5
		Glu7	1.6 ± 2.1

**Apo MBD4/SMD**			
MBD4 residues ΔGb		SMD residues ΔGb	
Met368	-1.4 ± 0.9	Met2	-1.5 ± 1.4
Thr369	-2.2 ± 1.6	Asp3	-2.3 ± 1.8
Cys370	-2.8 ± 1.5	Met4	-1.6 ± 1.2
Cys373	-1.3 ± 0.8	Ile6	-1.6 ± 1.1
Phe422	-1.1 ± 0.6		

**Apo MBD3/KER**			
MBD3 residues ΔGb		KER residues ΔGb	
Asn274	-2.4 ± 1.9	Arg3	-4.9 ± 2.9
Glu277	-1.8 ± 1.4	Tyr4	-2.4 ± 2.1
Asn278	-1.0 ± 1.1	Arg6	-4.5 ± 2.9

**Holo MBD3/KER**			
MBD3 residues ΔGb		KER residues ΔGb	
Cys271	-3.3 ± 2.3	Lys1	5.5 ± 3.2
Asn274	-3.3 ± 1.9	Glu2	-1.8 ± 0.6
Ile275	-1.2 ± 0.5	Arg3	-4.9 ± 2.6
Glu277	-1.2 ± 1.5		
Asn278	-2.2 ± 1.1		
Pro320	-4.4 ± 1.0		
Gly321	-1.2 ± 0.8		
Asn322	-2.0 ± 1.0		

Holo MBD4/KER – pose 1			
MBD3 residues ΔGb		KER residues ΔGb	
Cys373	-4.5 ± 1.1	Arg3	-5.4 ± 1.2
Met380	-3.1 ± 0.7	Thr4	-2.3 ± 0.9
Gln383	-1.3 ± 1.1	Arg6	-5.6 ± 2.0
Asp419	-1.7 ± 1.4		
Met420	-1.8 ± 0.8		

Holo MBD4/KER – pose 2			
MBD3 residues ΔGb		KER residues ΔGb	
Cys373	-4.1 ± 3.0	Lys1	-1.6 ± 1.6
Ser376	-2.0 ± 2.4	Arg3	-9.1 ± 1.7
Met420	-2.3 ± 1.7		
Phe422	-1.5 ± 0.7		

Table S4. Peptides' characterization.

Peptide	Sequence	MW (g/mole)	Purity (%)[1]
**Atox1 inhibitor** (SMD)	SMDMSIE	810.33	94 ± 1.5
**MBD4 inhibitor** (KER)	KERTSR	774.45	98 ± 1.0

[1] The error of purity was calculated based on three independent HPLC data

Table S5. Protein' sequence.

**Atox1**	10 20 30 40 50 60<br>MPKHEFSVDM TCGGCAEAVS RVLNKLGGVK YDIDLPNKKV CIESEHSMDT LLATLKKTGK<br><br>TVSYLGLE
**MBD3-4**	10 20 30 40 50 60<br>RPLSSANQNF NNSETLGHQG SHVVTLQLRI DGMHCKSCVL NIEENIGQLL GVQSIQVSLE<br><br>70 80 90 100 110 120<br>NKTAQVKYDP SCTSPVALQR AIEALPPGNF KVSLPDGAEG SGTDHRSSSS HSPGSPPRNQ<br><br>130 140 150 160 170 180<br>VQGTCSTTLI AIAGMTCASC VHSIEGMISQ LEGVQQISVS LAEGTATVLY NPSVISPEEL<br><br>190 200<br>RAAIEDMGFE ASVVSESCST NPLGNH
